# Supplementary material for: Evaluation of the global impacts of mitigation on persistent, bioaccumulative and toxic pollutants in marine fish
Source: PeerJ. 2016 Jan 28;4:e1573. doi: 10.7717/peerj.1573 (PMC4734435; doi:10.7717/peerj.1573)
Supplement: Supplemental Information 1 [file peerj-04-1573-s001.pdf]

Supplemental Material

Evaluation of the global impacts of mitigation on persistent, bioaccumulative and toxic pollutants in marine fish.

Lindsay T. Bonito, Amro Hamdoun, Stuart A. Sandin

Marine Biology Research Department, Scripps Institution of Oceanography, 9500 Gilman Drive, La Jolla, CA 92093-0202, USA

Table of Contents

Supplemental Figure 1: Regional Data Distribution.. **2**

Supplemental Figure 2: Habitat Data Distribution.. **3**

Supplemental Figure 3: Regional Temporal Analysis.. **4**

Supplemental Table 1: Sample Sizes and Data Distribution.. **5**

Supplemental Table 2: ANOVA Summary Table (Figure 2).. **6**

Supplemental Table 3: ANOVA Summary Table (Figure 3).. **6**

Supplemental Table 4: ANOVA Summary Table (Figure 4).. **7**

Supplemental Table 5: Linear Regression Summary (Figure 5).. **7**

Supplemental Table 6: Linear Regression Summary, Years 1990-2012.. **7**

Supplemental Table 7: Species List.. **8**

Supplemental Table 8: Seafood Database Reference List.. **26**

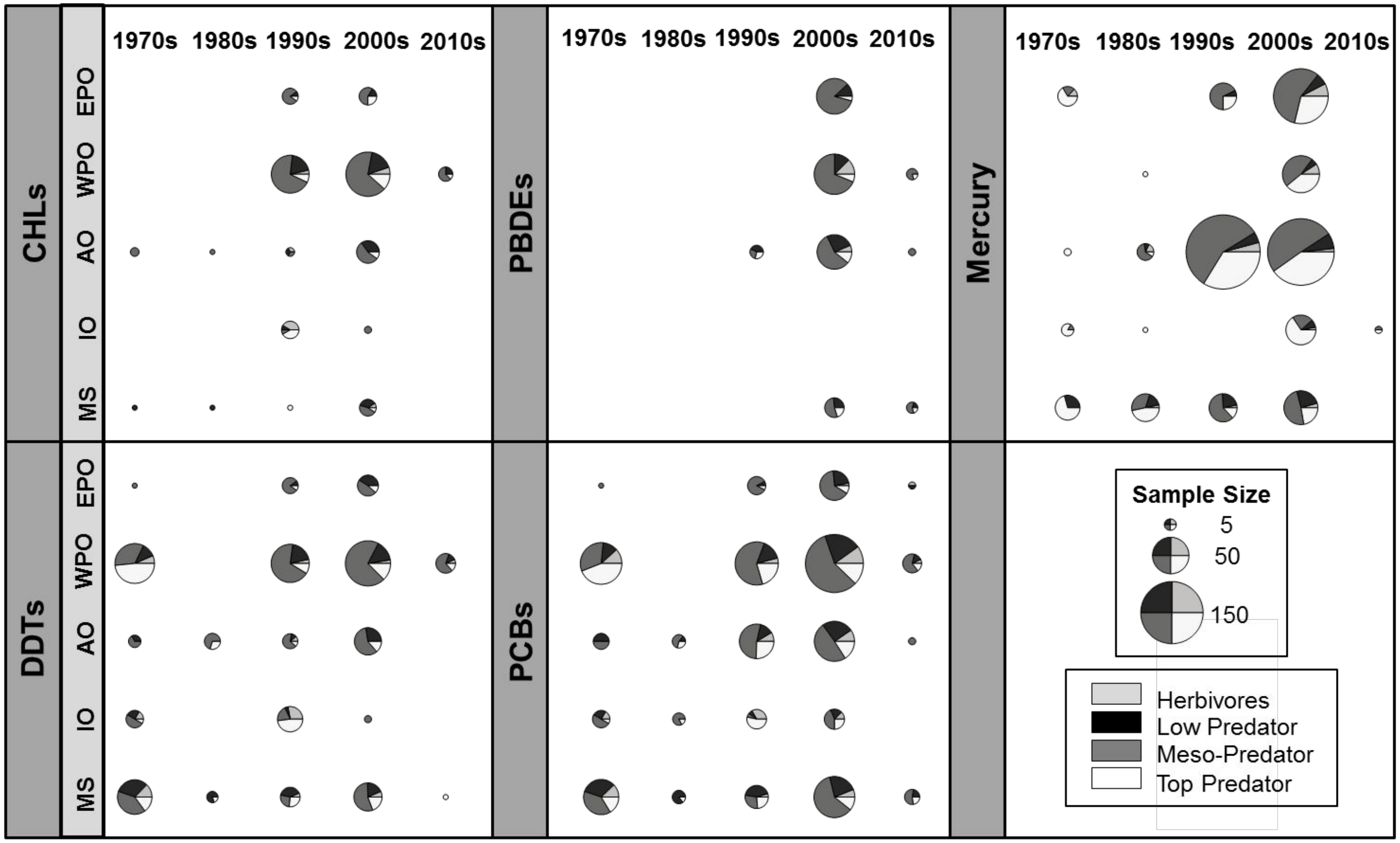

Supplemental Figure 1: Regional Data Distribution. Data distribution across pollutant groups, regions, and decades. Size of pie chart reflects number of data points included in analysis for each region. 5 global regions aggregated: EPO- East Pacific Ocean; WPO- West Pacific Ocean; AO- Atlantic Ocean; IO- Indian Ocean; MS- Mediterranean Sea.

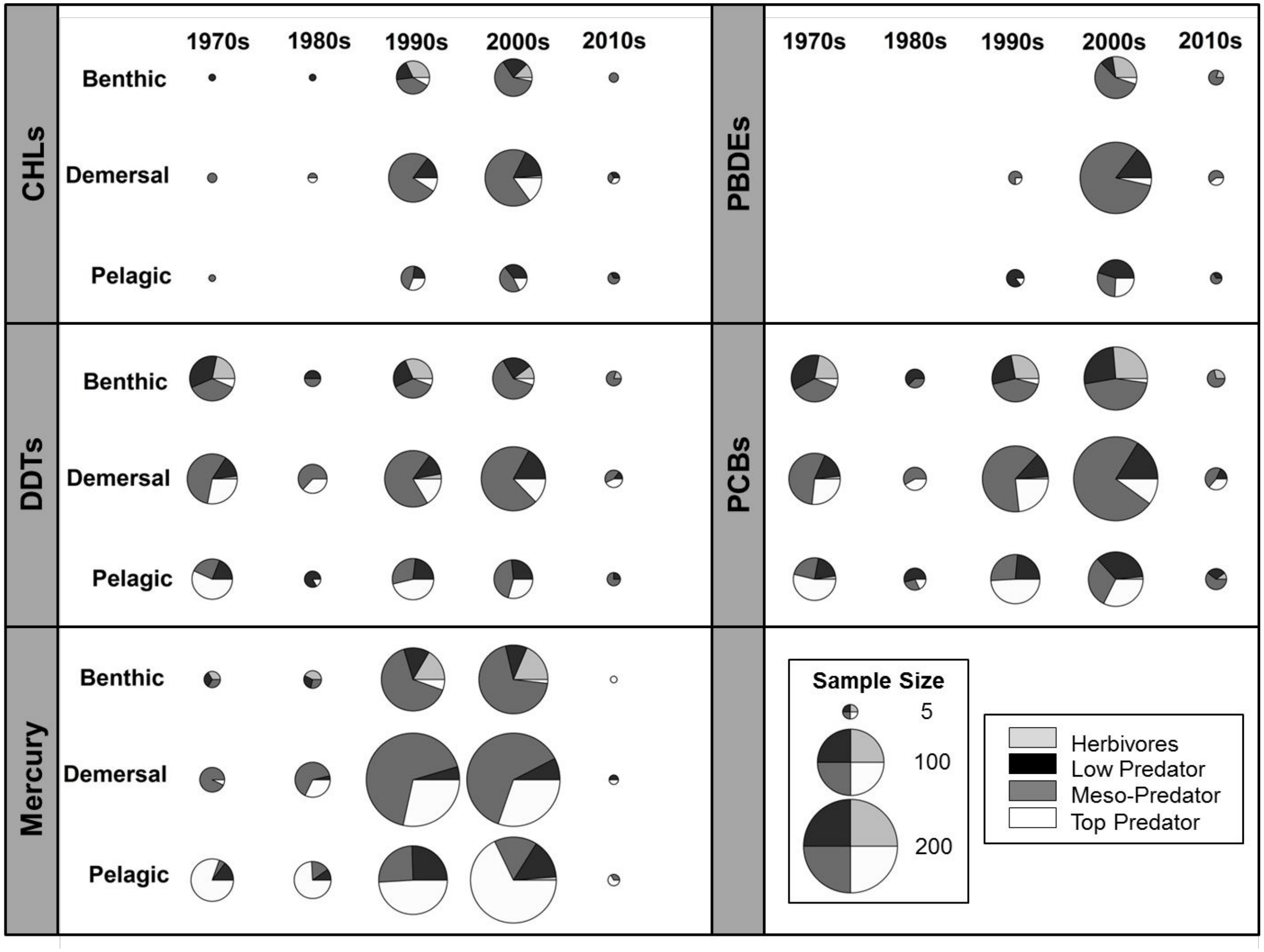

Supplemental Figure 2: Habitat Data Distribution. Data distribution across pollutant groups, feeding behavior, and decades. Size of pie chart reflects number of data points included in analysis for each feeding type per decade. Three feeding types were determined: benthic, demersal, and pelagic.

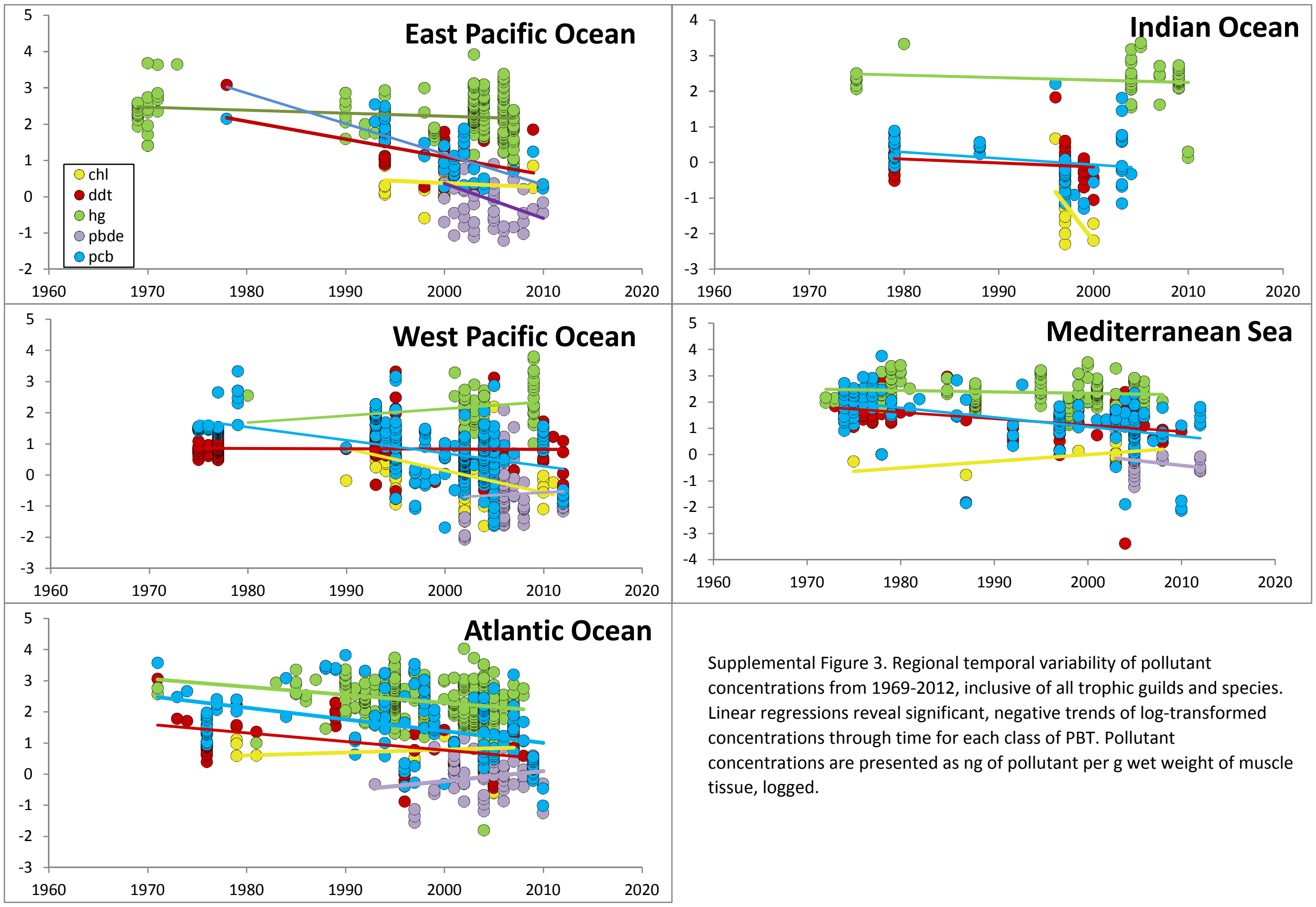

Supplemental Figure 3. Regional temporal variability of pollutant concentrations from 1969-2012, inclusive of all trophic guilds and species. Linear regressions reveal significant, negative trends of log-transformed concentrations through time for each class of PBT. Pollutant concentrations are presented as ng of pollutant per g wet weight of muscle tissue, logged.

Chem	Region	df	R^2	slope	F-stat	Pr(>F)	
CHL	EPO	20	0.01	-0.01186	0.2561	0.6183	
	WPO	138	0.2322	-0.07097	41.73	>0.001	*
	AO	26	0.01138	0.009214	0.2992	0.5891	
	IO	12	0.2699	-0.3451	4.436	0.05692	
	MS	12	0.2269	0.02531	3.523	0.08505	
DDT	EPO	26	0.1686	-0.04914	5.273	0.02997	
	WPO	205	0.000381	-0.0009433	0.07818	0.7801	
	AO	63	0.1821	-0.027785	14.03	0.000393	
	IO	37	0.03753	-0.011032	1.443	0.2373	
	MS	93	0.1522	-0.024387	16.7	>0.001	*
Hg	EPO	172	0.03252	-0.008151	5.782	0.01725	
	WPO	53	0.02444	0.02209	1.328	0.2544	
	AO	493	0.06569	-0.025769	34.66	>0.001	*
	IO	42	0.01668	-0.006703	0.7126	0.4034	
	MS	128	0.01139	-0.005485	1.475	0.2268	
PBDE	EPO	51	0.1441	-0.09622	8.586	0.005058	
	WPO	73	0.001761	0.0165	0.1287	0.7208	
	AO	52	0.03404	0.03177	1.832	0.1817	
	IO	-	-	-	-	-	
	MS	18	0.05117	-0.03922	0.9708	0.3375	
PCB	EPO	41	0.4963	-0.08425	40.4	>0.001	*
	WPO	284	0.2857	-0.041799	113.6	>0.001	*
	AO	136	0.1663	-0.037775	27.13	>0.001	*
	IO	47	0.05098	-0.01769	2.525	0.1188	
	MS	140	0.2467	-0.036032	45.85	>0.001	*

Supplemental Table 1. Summary of data distribution

	REGION	DECADE	H	P	MP	TP	TOTAL
CHL	EPO	1970	-	-	-	-	-
		1980	-	-	-	-	-
		1990	-	1	8	1	***10***
		2000	-	2	7	3	***12***
		2010	-	-	-	-	-
	WPO	1970	-	-	-	-	-
		1980	-	-	-	-	-
		1990	2	11	39	4	***56***
		2000	4	13	51	9	***77***
		2010	-	2	5	1	***8***
	AO	1970	-	-	3	-	***3***
		1980	-	-	1	-	***1***
		1990	1	1	1	-	***3***
		2000	-	7	11	2	***20***
		2010	-	-	-	-	-
	IO	1970	-	-	-	-	-
		1980	-	-	-	-	-
		1990	5	1	1	5	***12***
		2000	-	-	2	-	***2***
		2010	-	-	-	-	-
	MS	1970	-	1	-	-	***1***
		1980	-	1	-	-	***1***
		1990	-	-	-	1	***1***
		2000	1	4	5	1	***11***
		2010	-	-	-	-	-

	REGION	DECADE	H	P	MP	TP	TOTAL
DDT	EPO	1970	-	-	1	-	***1***
		1980	-	-	-	-	-
		1990	-	1	8	1	***10***
		2000	-	7	8	2	***17***
		2010	-	-	-	-	-
	WPO	1970	4	7	21	30	***62***
		1980	-	-	-	-	-
		1990	2	11	39	5	***57***
		2000	2	12	56	10	***80***
		2010	1	2	10	2	***15***
	AO	1970	-	2	4	-	***6***
		1980	-	-	7	3	***10***
		1990	1	1	6	1	***9***
		2000	-	8	17	4	***29***
		2010	-	-	-	-	-
	IO	1970	2	3	6	1	***12***
		1980	-	-	-	-	-
		1990	7	1	5	12	***25***
		2000	-	-	2	-	***2***
		2010	-	-	-	-	-
	MS	1970	6	15	19	7	***47***
		1980	-	4	-	1	***5***
		1990	1	6	4	4	***15***
		2000	2	6	17	6	***31***
		2010	-	-	-	1	***1***

PBDE	EPO	1970	-	-	-	-	-
		1980	-	-	-	-	-
		1990	-	-	-	-	-
		2000	-	6	42	2	***50***
		2010	-	-	-	-	-
	WPO	1970	-	-	-	-	-
		1980	-	-	-	-	-
		1990	-	-	-	-	-
		2000	8	8	44	4	***64***
		2010	-	-	4	1	***5***
	AO	1970	-	-	-	-	-
		1980	-	-	-	-	-
		1990	-	3	2	2	***7***
		2000	3	12	27	5	***47***
		2010	-	-	2	-	***2***
	IO	1970	-	-	-	-	-
		1980	-	-	-	-	-
		1990	-	-	-	-	-
		2000	-	-	-	-	-
		2010	-	-	-	-	-
	MS	1970	-	-	-	-	-
		1980	-	-	-	-	-
		1990	-	-	-	-	-
		2000	-	4	8	3	***15***
		2010	-	1	3	1	***5***

PCB	EPO	1970	-	-	1	-	***1***
		1980	-	-	-	-	-
		1990	-	1	11	1	***13***
		2000	1	8	22	3	***34***
		2010	1	1	-	-	***2***
	WPO	1970	8	8	22	30	***68***
		1980	-	-	-	-	-
		1990	3	11	44	15	***73***
		2000	13	27	76	16	***132***
		2010	1	2	9	2	***14***
	AO	1970	-	5	5	-	***10***
		1980	-	1	4	2	***7***
		1990	4	6	25	12	***47***
		2000	6	16	31	10	***63***
		2010	-	-	2	-	***2***
	IO	1970	2	3	6	1	***12***
		1980	-	-	5	1	***6***
		1990	5	1	1	8	***15***
		2000	2	3	7	4	***16***
		2010	-	-	-	-	-
	MS	1970	6	16	19	8	***49***
		1980	-	6	-	1	***7***
		1990	1	9	6	5	***21***
		2000	4	15	40	7	***66***
		2010	-	2	5	2	***9***

Hg	EPO	1970	2	-	3	10	***15***
		1980	-	-	-	-	-
		1990	-	2	19	7	***28***
		2000	9	8	67	34	***118***
		2010	-	-	-	-	-
	WPO	1970	-	-	-	-	-
		1980	-	-	-	1	***1***
		1990	-	-	-	-	-
		2000	5	3	25	21	***54***
		2010	-	-	-	-	-
	AO	1970	-	-	-	2	***2***
		1980	2	1	7	1	***11***
		1990	9	10	124	73	***216***
		2000	3	13	88	70	***174***
		2010	-	-	-	-	-
	IO	1970	-	-	1	5	***6***
		1980	-	-	-	1	***1***
		1990	-	-	-	-	-
		2000	1	3	8	23	***35***
		2010	-	-	1	1	***2***
	MS	1970	-	7	-	17	***24***
		1980	1	5	10	14	***30***
		1990	1	7	19	4	***31***
		2000	2	11	22	10	***45***
		2010	-	-	-	-	-

Regions: *EPO-East Pacific Ocean; WPO- West Pacific Ocean; AO- Atlantic Ocean; IO- Indian Ocean; MS- Mediterranean Sea*

Trophic Levels: *H- Herbivore; P- Primary Predator; MP- Middle Predator; TP- Top Predator*

Supplemental Table 2. Summary data from analyses presented in Figure 2

	df	Pr(>F)	
**Hg**	4, 790	0.753	
**CHL**	4, 207	p < 0.001	*
**DDT**	4, 275	0.625	
**PBDE**	3, 198	0.697	
**PCB**	4, 483	p < 0.0001	*

Supplemental Table 3. Summary data from analyses presented in Figure 3

		df	Pr(>F)		Region N-values				
					EPO	*WPO*	*AO*	*IO*	*MS*
**Herbivores**	*CHL*	3, 9	0.322		-	6	1	5	1
	DDT	3, 11	0.032		-	4	1	7	3
	Hg	4, 32	0.008	**	9	5	19	1	3
	PBDE	1, 12	0.333		-	11	3	-	-
	PCB	4, 36	0.288		2	17	10	7	5
**Low Predators**	*CHL*	4, 37	0.341		3	26	8	1	4
	DDT	4, 49	0.476		8	25	8	1	12
	Hg	4, 60	0.457		10	3	31	3	18
	PBDE	3, 32	0.374		8	8	15	-	5
	PCB	4, 94	0.309		8	40	21	4	26
**Mid Predators**	*CHL*	4, 125	0.123		19	95	12	3	5
	DDT	4, 159	0.311		16	101	21	7	19
	Hg	4, 412	0.165		86	25	256	9	41
	PBDE	3, 129	0.153		43	50	29	-	11
	PCB	4, 260	0.161		29	129	55	8	44
**Top Predators**	*CHL*	4, 22	0.11		4	13	3	5	2
	DDT	4, 42	0.177		3	15	6	12	11
	Hg	4, 271	0.449		41	21	176	24	14
	PBDE	3, 15	0.261		2	6	7	-	4
	PCB	4, 78	0.105		3	32	22	12	14

Supplemental Table 4. Summary data from analyses presented in Figure 4

		df	Pr(>F)	
**Hg**	*Trophic*	3, 791	p < 0.001	**
	Habitat	2, 792	p< 0.001	**
**CHL**	*Trophic*	3, 208	0.494	
	Habitat	2, 209	0.311	
**DDT**	*Trophic*	3, 276	0.142	
	Habitat	2, 277	0.352	
**PBDE**	*Trophic*	3, 198	0.087	
	Habitat	2, 199	0.155	
**PCB**	*Trophic*	3, 484	0.466	
	Habitat	2, 485	0.222	

Supplemental Table 5. Summary data from analyses presented in Figure 5

	df	R^2	slope	F-stat	Pr(>F)	
**CHL**	229	0.02	-0.02	5.121	0.02	*
**DDT**	488	0.06	-0.02	31.17	p < 0.001	*
**Hg**	997	0.03	-0.01	27.6	p < 0.001	*
**PBDE**	216	0.01	-0.03	4.155	0.04	*
**PCB**	722	0.17	-0.04	151.4	p < 0.001	*

Supplemental Table 6: Subset of summary data from analyses presented in Figure 5 (years 1990-2012)

	df	R^2	slope	F-stat	Pr(>F)	
**CHL**	223	0.02	-0.02	4.911	0.03	*
**DDT**	318	0.00	-0.004	0.245	0.62	
**Hg**	893	0.02	-0.015	15.51	p < 0.001	*
**PBDE**	216	0.02	-0.03	4.155	0.042	*
**PCB**	542	0.09	-0.06	52.36	p < 0.001	*

Supplemental Table 7. Summary of species information & attributes in database

Common Name	Scientific Name	Trophic Level	Trophic Guild	Habitat
wahoo	Acanthocybium solandri	4.4 ± 0.8	TP	Pelagic
yellowfin goby	Acanthogobius flavimanus	3.3 ± 0.4	P	Benthic
St. Paul's fingerfin	Acantholatris monodactylus	3.5 ± 0.41	MP	Demersal
yellowfin bream	Acanthopagrus australis	3.1 ± 0.4	MP	Demersal
picnic seabream	Acanthopagrus berda	3.5 ± 0.6	MP	Benthic
yellowfin seabream	Acanthopagrus latus	3.2 ± 0.5	P	Demersal
black seabream	Acanthopagrus schlegel	3.2 ± 0.45	P	Demersal
yellowfin surgeonfish	Acanthurus xanthopterus	2.9 ± 0.36	H	Benthic
goby	Acentrogobius janthinopterus	2.5 ± 0.5	H	Benthic
shortnose sturgeon	Acipenser brevirostrum	3.3 ± 0.39	MP	Benthic
Russian sturgeon	Acipenser gueldenstaedtii	3.1 ± 0.3	MP	Benthic
ship sturgeon	Acipenser nudiventris	3.3 ± 0.45	MP	Benthic
Persian sturgeon	Acipenser persicus	3.7 ± 0.59	MP	Demersal
stellate sturgeon	Acipenser stellatus	3.5 ± 0.2	MP	Demersal
white sturgeon	Acipenser transmontanus	3.2 ± 0.4	MP	Demersal
glowbelly	Acropoma japonica	3.3 ± 0.4	P	Demersal
giant grenadier	Albatrossia pectoralis	4.3 ± 0.8	P	Demersal
bonefish	Albula vulpes	3.7 ± 0.3	TP	Benthic
rainbow sculpin	Alcichthys alcicornis	3.6 ± 0.6	MP	Demersal
shrimp scad	Alepes djedaba	3.3 ± 0.47	MP	Pelagic
Risso's smoothhead	Alepocephalus rostratus	3.5 ± 0.5	MP	Pelagic
pelagic thresher shark	Alopias pelagicus	4.5 ± 0.66	TP	Pelagic
**thresher sharks**	**Alopias spp.**	**4.5 ± 0**	**TP**	**Pelagic**
bigeye thresher shark	Alopias superciliosus	4.5 ± 0.8	TP	Pelagic
thresher shark	Alopias vulpinus	4.5 ± 0	TP	Pelagic
allis shad	Alosa alosa	3.6 ± 0.53	MP	Pelagic
alewife	Alosa pseudoharengus	3.5 ± 0.5	P	Demersal
glass perchlet	Ambassis vachellii	2.5 ± 0.5	H	Demersal
gobies	Amblychaeturichthys sciistius	3.3 ± 0.4	P	Demersal
starry ray	Amblyraja radiata	4.2 ± 0.3	TP	Benthic
lesser sand eel	Ammodytes tobianus	3.1 ± 0.3	P	Demersal
Northern wolffish	Anarhichas denticulatus	3.8 ± 0.46	MP	Demersal
Atlantic wolffish	Anarhichas lupus	3.8 ± 0.46	MP	Demersal
spp..otted wolffish	Anarhichas minor	3.5 ± 0.4	MP	Benthic
frys	Anchoa choerostoma	3.3 ± 0.4	P	Pelagic
anchovy	Anchoa mitchilli	3.5 ± 0.5	P	Demersal
eel	Anguilla anguilla	3.5 ± 0.6	MP	Benthic
Japanese eel	Anguilla japonica	3.6 ± 0.51	MP	Demersal
American eel	Anguilla rostrata	3.7 ± 0.6	MP	Demersal
eel	Anguilla spp	3.7 ± 0.6	MP	Demersal
chacuna gizzardshad	Anodontostoma chacunda	2.8 ± 0.8	H	Benthic
sablefish	Anoplopoma fimbria	3.8 ± 0.6	MP	Demersal
black scabbard	Aphanopus carbo	4.5 ± 0.77	TP	Pelagic
cardinalfish	Apogon hyalosoma	2.5 ± 0.5	H	Benthic

cardinal fish; Indian perch	Apogon lineatus	3.7 ± 0.6	P	Benthic
sheepshead	Archosargus probatocephalus	3.5 ± 0.53	MP	Benthic
Western Atlantic seabream	Archosargus rhomboidalis	2.9 ± 0.1	P	Benthic
polar cod	Arctogadus glacialis	3.8 ± 0.61	MP	Demersal
small mouth argentine	Argentina kagoshimae	3.4 ± 0.4	MP	Benthic
king soldier bream	Argyrops spinifer	4.5 ± 0.8	TP	Benthic
conger eel	Ariosoma shiroanago major	4 ± 0.67	TP	Demersal
hardhead catfish	Arius felis	3.3 ± 0.6	MP	Demersal
giant catfish	Arius serratus	3.1 ± 0.3	P	Benthic
Chinese catfish	Arius sinensis	3.9 ± 0.66	MP	Benthic
giant sea catfish	Arius thalassinus	3.1 ± 0.3	P	Demersal
scaldfish	Arnoglossus laterna	3.6 ± 0.5	MP	Benthic
red gurnard	Aspitrigla cuculus	3.9 ± 0.6	MP	Benthic
jellynose fishes	Ateleopus japonicus	4.2 ± 0.73	MP	Demersal
sand smelt	Atherina presbyter	3.7 ± 0.43	P	Pelagic
topsmelt	Atherinops affinis	2.8 ± 0.28	H	Pelagic
jacksmelt	Atherinopsis californiensis	3.1 ± 0.5	P	Pelagic
cleftbelly trevally	Atropus atropos	3.6 ± 0.52	MP	Pelagic
Japanese thread sail fish	Aulopus japonicus	4 ± 0.66	MP	Demersal
frigate mackerel	Auxis thazard	4.3 ± 0.7	TP	Pelagic
gafftopsail sea catfish	Bagre marinus	3.5 ± 0.5	MP	Demersal
silver perch	Bairdiella chrysoura	3.2 ± 0.4	P	Benthic
grey triggerfish	Balistes capriscus	4.1 ± 0.2	MP	Benthic
titan triggerfish	Balistoides viridescens	3.3 ± 0.44	P	Benthic
**rattail fishes**	**Bathygadus spp.**	**3.2 ± 0.3**	**P**	**Demersal**
spinytail skate	Bathyraja spinicauda	4.4 ± 0.6	TP	Benthic
**temperate perches**	**Bathysphryaenops spp.**	**3.9 ± NA**	**MP**	**Demersal**
deepwater flatheads	Bembras japonica	3.6 ± 0.6	MP	Demersal
alfonsino	Beryx splendens	4.3 ± 0.2	MP	Demersal
goldenspot hogfish	Bodianus perditio	4.1 ± 0	MP	Benthic
green humphead parrotfish	Bolbometopon muricatum	2.7 ± 0.41	H	Benthic
bogue	Boops boops	2.8 ± 0	P	Benthic
salema	Boops salpa	2 ± 0	H	Benthic
arctic cod	Boreogadus saida	3.1 ± 0.3	P	Pelagic
soft eelpout	Bothrocara molle	3.4 ± 0.5	MP	Demersal
**flounders**	**Bothus spp.**	**4.4 ± 0**	**MP**	**Benthic**
spotted sole	Brachirius orientalis	3.5 ± 0.37	MP	Benthic
Atlantic pomfret	Brama brama	4.1 ± 0.64	MP	Pelagic
menhaden	Brevoortia patronus	2.2 ± 0.07	P	Pelagic
yellowfin menhaden	Brevoortia smithi	2.3 ± 0	P	Pelagic
Atlantic menhaden	Brevoortia tyrannus	2.3 ± 0.2	P	Pelagic
tusk	Brosme brosme	3.9 ± 0.3	MP	Benthic
grenadiers	Caelorinchus jordani	3.5 ± 0.5	MP	Benthic
spearnose grenadier	Caelorinchus multispinulosus	3.6 ± 0.5	MP	Benthic
jolthead porgy	Calamus bajonado	3.5 ± 0.2	MP	Demersal
saucereye porgy	Calamus calamus	3.5 ± 0.2	MP	Demersal
dragonet	Callionymus lyra	3.3 ± 0.83	P	Demersal
**jacks**	**Carangidae spp.**	**4.4 ± 0.8**	**TP**	**Pelagic**

yellow jack	Carangoides bartholomaei	4.5 ± 0.8	TP	Pelagic
malabar trevally	Carangoides malabaricus	4.4 ± 0.5	TP	Pelagic
yellow jack	Caranx bartholomaei	4.5 ± 0.8	TP	Pelagic
Pacific crevalle jack; crevalle jack	Caranx caninus	3.7 ± 0.56	MP	Pelagic
blue runner	Caranx crysos	4.4 ± 0.8	MP	Pelagic
blue runner	Caranx fusus	4.4 ± 0.8	MP	Pelagic
crevalle jack	Caranx hippos	3.5 ± 0.5	MP	Demersal
giant trevally	Caranx ignobilis	4.2 ± 0.7	TP	Pelagic
malabar trevally	Caranx malabaricus	4.4 ± 0.5	TP	Pelagic
bluefin trevally	Caranx melampygus	4.5 ± 0.8	TP	Pelagic
bar jack	Caranx ruber	4.3 ± 0.1	MP	Demersal
blacktip trevally	Caranx sem	3.7 ± 0.56	MP	Pelagic
bigeye trevally	Caranx sexfasciatus	4.5 ± 0.8	MP	Pelagic
blacknose shark	Carcharhinus acronotus	4.4 ± 0.5	MP	Pelagic
spinner shark	Carcharhinus brevipinna	4.2 ± 0.6	MP	Pelagic
silky shark	Carcharhinus falciformis	4.5 ± 0.6	TP	Pelagic
dusky shark (Galapagos)	Carcharhinus galapagensis	4.2 ± 0.4	TP	Pelagic
finetooth shark	Carcharhinus isodon	4.2 ± 0.7	MP	Pelagic
bull shark	Carcharhinus leucas	4.3 ± 0.7	TP	Pelagic
blacktip shark	Carcharhinus limbatus	4.2 ± 0.7	MP	Pelagic
dusky shark	Carcharhinus obscurus	4.3 ± 0.2	TP	Demersal
Caribbean reef shark	Carcharhinus perezi	4.5 ± 0.8	TP	Pelagic
sandbar shark	Carcharhinus plumbeus	4.5 ± 0	TP	Demersal
night shark	Carcharhinus signatus	4.5 ± 0.4	TP	Pelagic
silky shark	Carcharinus falciformis	4.5 ± 0.6	TP	Pelagic
great white shark	Carcharodon carcharias	4.5 ± 0.4	TP	Pelagic
sakebikunin	Careproctus rastrinus	3.3 ± 0.49	MP	Demersal
ocean whitefish	Caulolatilus princeps	3.9 ± 0.6	MP	Benthic
rock seabass	Centopristis philadelphica	3.9 ± 0.6	MP	Demersal
gulper shark	Centrophorus granulosus	4.1 ± 0.7	TP	Pelagic
armed snook	Centropomus armatus	3.9 ± 0.7	MP	Demersal
blackfin snook	Centropomus medius	4 ± 0.65	MP	Demersal
black snook	Centropomus nigrescens	4.2 ± 0.8	MP	Demersal
fat snook	Centropomus parallelus	4.2 ± 0.74	TP	Demersal
common snook	Centropomus undecimalis	4.4 ± 0.8	TP	Demersal
rock sea bass	Centropristis philadelphica	3.9 ± 0.6	MP	Demersal
black seabass	Centropristis striata	3.9 ± 0.2	MP	Demersal
striped bass	Centropristis striate	4 ± 0.6	MP	Demersal
blue spotted grouper	Cephalopholis argus	4.5 ± 0.8	TP	Demersal
chocolate hind	Cephalopholis boenak	4.2 ± 0.7	TP	Demersal
coney	Cephalopholis fulva	4.1 ± 0.7	TP	Demersal
tomato hind	Cephalopholis sonnerati	3.8 ± 0.6	MP	Demersal
red bandfish	Cepola macrophthalma	3.1 ± 0.23	P	Demersal
Warming's lantern fish	Ceratoscopelus warmingi	3.4 ± 0.53	MP	Pelagic
spade	Chaetodipterus faber	4.5 ± 0	MP	Benthic
catfish	Chaeturichthys stigmatias	3.3 ± 0.4	MP	Demersal
mackerel icefish	Champsocephalus gunnari	3.2 ± 0.4	MP	Pelagic

milkfish	Chanos chanos	2 ± 0.1	H	Benthic
lefteye flounder	Chascanopsetta lugubris	3.5 ± 0.37	MP	Benthic
sea toads	Chaunax abei	4.3 ± 0.6	TP	Demersal
floral wrasse	Cheilinus chlorourus	3.4 ± 0.5	P	Benthic
redbreasted wrasse	Cheilinus fasciatus	3.4 ± 0.4	P	Benthic
tripletail wrasse	Cheilinus trilobatus	3.5 ± 0.5	P	Benthic
tub gurnard	Chelidonichthys lucerna	3.7 ± 0.6	MP	Demersal
thicklip grey mullet	Chelon labrosus	2.6 ± 0.32	H	Demersal
bamboo shark	Chiloscyllium plagiosum	4 ± 0.67	MP	Benthic
ghostshark	Chimaera monstrosa	3.5 ± 0	MP	Benthic
crocodile icefish	Chionodraco hamatus	3.7 ± 0.6	MP	Demersal
dorab wolfherring	Chirocentrus dorab	4.5 ± 0.8	TP	Pelagic
greeneyes	Chlorophthalmus acutifrons	4 ± 0.67	MP	Demersal
greeneyes	Chlorophthalmus albatrossis	4.1 ± 0.69	MP	Demersal
doublespotted queenfish	Chorinemus lysan	4.5 ± 0.8	TP	Pelagic
fivebeard rockling	Ciliata mustela	3.5 ± 0.6	MP	Demersal
speckled sanddab	Citharichthys stigmaeus	3.4 ± 0.52	MP	Demersal
spotted founder	Citharus linguatula	4 ± 0.65	MP	Demersal
spotted flounder	Citharus macrolepidotus	4 ± 0.65	MP	Demersal
arrow goby	Clevelandia ios	3.1 ± 0.42	MP	Benthic
shad	Clupanodon punctatus	2.9 ± 0.22	H	Benthic
Baltic herring	Clupea harengus	3.2 ± 0.4	P	Pelagic
white sea herring	Clupea pallasi marisalbi	3.1 ± 0.3	P	Pelagic
demon grenadier	Coelorinchus gilberti	3.7 ± 0.5	MP	Benthic
spearsnouted grenadier	Coelorinchus labiatus	4 ± 0.65	MP	Demersal
longarm grenadier	Coelorinchus macrochir	3.8 ± 0.62	MP	Demersal
ungaro	Coelorinchus mediterraneus	3.5 ± 0.5	MP	Demersal
unicorn grendarier	Coelorinchus productus	3.5 ± 0.5	MP	Demersal
croaker	Collichthys niveatus	3.4 ± 0.45	MP	Demersal
Pacific saury	Cololabis saira	3.7 ± 0.44	MP	Pelagic
**conger**	**Conger conger**	**4.3 ± 0.8**	**TP**	**Demersal**
garden eel	Conger myriaster	4 ± 0.68	MP	Demersal
argentine conger	Conger orbignianus	3.7 ± 0.59	MP	Demersal
whitefish	Coregonus lavaretus	3.1 ± 0.4	P	Demersal
dolphinfish; mahi mahi	Coryphaena hippurus	4.4 ± 0	TP	Pelagic
Pacific grenadier	Coryphaenoides acrolepis	3.8 ± 0.54	MP	Demersal
grenadier	Coryphaenoides armatus	3.6 ± 0.52	MP	Benthic
Mediterranean grenadier	Coryphaenoides mediterraneus	3.4 ± 0.41	MP	Benthic
largenose grenadier	Coryphaenoides nasutus	3.6 ± 0.5	MP	Demersal
roundnose grenadier	Coryphaenoides rupestris	3.5 ± 0.49	MP	Demersal
gold sculpin	Cottiusculus schmidti	3.2 ± 0.4	P	Demersal
karanteen seabream	Crenidens crenidens	2.8 ± 0.29	H	Demersal
two-spot surgeonfish	Ctenochaetus binotatus	2 ± 0	H	Benthic
striated surgeonfish	Ctenochaetus striatus	2 ± 0	H	Benthic
lumpfish	Cyclopterus lumpus	3.9 ± 0	MP	Pelagic
shiner surfperch	Cymatogaster aggregata	3 ± 0.31	P	Demersal
crocodile fish	Cymbacephalus beauforti	4.5 ± 0	MP	Demersal
four-lined tonguesole	Cynoglossus bilineatus	3.5 ± 0.37	MP	Benthic

long tongue sole	Cynoglossus lingua	3.5 ± 0.37	MP	Benthic
large-scaled tongue sole	Cynoglossus macrolepidotus	3.5 ± 0.4	MP	Benthic
sole	Cynoglossus robustus	3.5 ± 0.4	MP	Benthic
halfsmooth tonguesole	Cynoglossus semilaevis	3.7 ± 0.6	MP	Benthic
four-lined tonguesole	Cynoglossus sidensis	3.5 ± 0.37	MP	Benthic
**tonguefishes**	**Cynoglossus spp.**	**3.5 ± 0.4**	**MP**	**Demersal**
acoupa weakfish	Cynoscion acoupa	4.1 ± 0.7	MP	Demersal
sand weakfish	Cynoscion arenarius	4.3 ± 0.8	MP	Demersal
stripped weakfish	Cynoscion guatucupa	4.2 ± 0.7	MP	Demersal
spotted sea trout	Cynoscion nebulosus	4 ± 0.66	MP	Demersal
silver perch	Cynoscion nothus	4 ± 0.7	P	Demersal
weakfish	Cynoscion regalis	3.8 ± 0.4	MP	Demersal
striped weakfish	Cynoscion striatus	3.9 ± 0.6	MP	Demersal
green weakfish	Cynoscion virescens	4 ± 0.7	MP	Demersal
flying fish	Cypselurus heterurus	3.4 ± 0.45	P	Pelagic
stingray	Dasyatis akajei	3.8 ± 0.58	MP	Benthic
Southern stingray	Dasyatis americana	3.5 ± 0.6	MP	Benthic
whiptail stingray	Dasyatis brevis	3.8 ± 0.6	MP	Benthic
longtail stingray	Dasyatis longus	3.5 ± 0.37	MP	Benthic
Atlantic stingray	Dasyatis sabina	3.5 ± 0.42	MP	Benthic
bluntnose stingray	Dasyatis say	3.5 ± 0.6	MP	Benthic
pale-edged stingray	Dasyatis zugei	3.5 ± 0.57	MP	Benthic
spinyhead sculpin	Dasycottus setiger	3.5 ± 0.5	MP	Demersal
sting ray	Dasytis sabina	3.5 ± 0.42	MP	Demersal
scorpion fish	Deania calcea	4.2 ± 0.7	TP	Demersal
mackerel scad	Decapterus macarellus	4 ± 0.2	MP	Pelagic
blue scad	Decapterus maruadsi	3.4 ± 0.45	MP	Pelagic
Indian scad	Decapterus russelli	3.7 ± 0.6	MP	Demersal
goatee croaker	Dendrophysa russelii	3.5 ± 0.5	MP	Demersal
yellowback; crimson seabream	Dentex tumifrons	3.8 ± 0.56	MP	Demersal
painted sweetlips	Diagramma pictum	3.7 ± 0.2	MP	Benthic
lantern fish	Diaphus chrysorhynchus	3.1 ± 0.3	P	Benthic
lantern fish	Diaphus suborbitalis	3.1 ± 0.3	P	Pelagic
California headlightfish	Diaphus theta	3.1 ± 0.25	P	Demersal
lantern fish	Diaphus watasei	3.2 ± 0.3	P	Benthic
Irish mojarra	Diapterus auratus	2.4 ± 0.9	H	Benthic
Peruvian mojarra	Diapterus peruvianus	3.7 ± 0.2	MP	Benthic
striped mojarra	Diapterus plumieri	2.2 ± 0	H	Benthic
sea bass	Dicentrarchus labrax	3.8 ± 0.6	MP	Demersal
sand perch	Diplectrum formosum	4.5 ± 0.8	MP	Demersal
annular seabream	Diplodus annualris	3.4 ± 0.4	MP	Benthic
sharpsnout seabream	Diplodus puntazzo	2.9 ± 0.4	H	Benthic
white seabream	Diplodus sargus	3 ± 0.3	P	Demersal
common two-banded seabream	Diplodus vulgaris	3.2 ± 0.4	P	Demersal
Patagonian toothfish	Dissostichus eleginoides	4 ± 0.68	TP	Demersal
blackthroat seaperch	Doederleinia berycoides	3.9 ± 0.56	MP	Demersal
rainbow runner	Elagatis bipinnulata	4.3 ± 0	MP	Demersal
navaga	Eleginus navaga	4.2 ± 0.73	TP	Demersal

fourfinger threadfin	Eleutheronema tetradactylum	4.4 ± 0.8	TP	Demersal
Pacific layfish	Elops affinis	4.1 ± 0.7	MP	Demersal
mullet	Elops saurus	4 ± 0.7	MP	Demersal
anchovy	Engraulis encrasicolus	3.1 ± 0.45	P	Pelagic
Japanese anchovy	Engraulis japonica	3.1 ± 0.1	P	Pelagic
northern anchovy	Engraulis mordax	3 ± 0.3	P	Pelagic
oblique-jaw thryssa	Engraulis purava	3.5 ± 0.5	MP	Pelagic
orbfish	Ephippus orbis	4 ± 0.61	TP	Benthic
sling-jaw wrasse	Epibulus insidiator	3.8 ± 0.7	MP	Benthic
areolate grouper	Epinephelus areolatus	3.6 ± 0.6	MP	Demersal
chocolate hind	Epinephelus boenack	4.2 ± 0.7	TP	Demersal
whitespotted grouper	Epinephelus coeruleopunctatus	3.7 ± 0.5	MP	Demersal
banded grouper	Epinephelus coioides	3.9 ± 0.7	MP	Demersal
graysby	Epinephelus cruentatus	4.3 ± 0.6	TP	Demersal
speckled hind	Epinephelus drummondhayi	4 ± 0.6	MP	Demersal
banded reef cod	Epinephelus fasciatus	3.7 ± 0.6	MP	Demersal
yellowedge grouper	Epinephelus flavolimbatus	3.8 ± 0.61	MP	Benthic
red hind	Epinephelus guttatus	3.8 ± 0.3	MP	Demersal
Atlantic goliath grouper	Epinephelus itajara	4.1 ± 0	TP	Demersal
highfin grouper	Epinephelus maculatus	3.9 ± 0.7	MP	Demersal
dusky grouper	Epinephelus marginatus	3.7 ± 0.6	MP	Demersal
honeycomb grouper	Epinephelus merra	3.8 ± 0.6	MP	Demersal
red grouper	Epinephelus morio	3.5 ± 0.5	MP	Demersal
snowy grouper	Epinephelus niveatus	4 ± 0.58	MP	Demersal
sixbar grouper	Epinephelus sexfasciatus	3.8 ± 0.6	MP	Demersal
jack-knifefish	Equetus lanceolatus	3.4 ± 0.5	P	Demersal
splendid ponyfish	Equula splendens	2.9 ± 0.38	H	Benthic
bird sculpin	Ereunias grallator	3.6 ± 0.6	MP	Demersal
deep water red snapper	Etelis oculatus	4.2 ± 0.57	MP	Demersal
bonga shad	Ethmalosa fimbriata	2.5 ± 0	H	Pelagic
blackbelly lanternshark	Etmopterus lucifer	4.2 ± 0.5	MP	Pelagic
chromide	Etroplus suratensis	2.5 ± 0.5	H	Benthic
splendid ponyfish	Eubleekeria splendens	2.9 ± 0.38	H	Benthic
mojarra	Eucinostomus gula	2.7 ± 0.1	H	Benthic
kawakawa	Euthynnus affinis	4.5 ± 0.8	TP	Pelagic
little tunny	Euthynnus alletteratus	4.5 ± 0.8	TP	Pelagic
grey gurnard	Eutrigla gurnardus	3.6 ± 0.6	MP	Demersal
**cornetfishes**	**Fistularia spp.**	**4.5 ± 0.8**	**TP**	**Demersal**
mummichog	Fundulus heteroclitus	3.6 ± 0.48	MP	Demersal
silvery pout	Gadiculus argenteus	3.6 ± 0.3	P	Benthic
Pacific cod	Gadus macrocephalus	4 ± 0.7	MP	Benthic
whiting	Gadus merlangus	3.6 ± 0.4	MP	Demersal
cod	Gadus morhua	4.4 ± 0.8	TP	Demersal
white sea cod	Gadus morhua marisalbi	3.6 ± 0.6	MP	Demersal
greenland cod	Gadus ogac	3.6 ± 0.6	MP	Demersal
blue whiting	Gadus poutassou	4 ± 0.7	MP	Demersal
**cods**	**Gadus spp.**	**3.6 ± 0.4**	**MP**	**Demersal**
catfish	Galeichthys felis	3.3 ± 0.6	P	Benthic

tiger shark	Galeocerdo cuvier	4.5 ± 0.7	TP	Pelagic
three-spined stickleback	Gasterosteus aculeatus	3.5 ± 0.5	MP	Benthic
Guri sea catfish	Genidens genidens	3.5 ± 0.4	MP	Demersal
white croaker	Genyonemus lineatus	3.4 ± 0.49	MP	Benthic
kingclip	Genypterus capensis	4.4 ± 0.61	TP	Demersal
common silverbiddy	Gerres argyreus	3.1 ± 0.2	P	Benthic
whipfin mojarra	Gerres filamentosus	3.3 ± 0.4	P	Benthic
parore	Girella tricuspidata	2 ± 0	H	Benthic
argentines	Glossanodon semifasciatus	3.1 ± 0.34	P	Benthic
witch	Glyptocephalus cynoglossus	3.1 ± 0.3	MP	Benthic
Japanese cusk	Glyptophidium japonicum	3.5 ± 0.6	MP	Benthic
black goby	Gobius niger	3.2 ± 0.4	MP	Benthic
**gobies**	**Gobius spp.**	**3.3 ± 0.39**	**MP**	**Demersal**
morwongs	Goniistius quadricornis	2.7 ± 0.25	H	Benthic
ploughfish	Gymnodraco acuticeps	4.5 ± 0.8	TP	Demersal
Nichol's lanternfish	Gymnoscopelus nicholsi	3.4 ± 0.5	P	Pelagic
giant moray	Gymnothorax javanicus	3.9 ± 0.64	TP	Demersal
kidako moray eels	Gymnothorax kidako	4.5 ± 0.61	TP	Demersal
California butterfly ray	Gymnura marmorata	4 ± 0.65	MP	Benthic
smooth butterfly ray	Gymnura micrura	3.6 ± 0.53	MP	Demersal
white margate	Haemulon album	3.3 ± 0.1	MP	Benthic
caesar grunt	Haemulon carbonarium	3.3 ± 0.5	MP	Demersal
white grunt	Haemulon plumieri	3.8 ± 0	MP	Demersal
bluestriped grunt	Haemulon sciurus	3.5 ± 0.2	MP	Demersal
greybar grunt	Haemulon sexfaciatum	4.2 ± 0.73	MP	Benthic
yellowstripe grunt	Haemulopsis axillaris	3.4 ± 0.6	MP	Demersal
narrowmouthed catshark	Halaculurus bivius	3.8 ± 0.5	MP	Demersal
scaled herring	Harengula jaguana	3.4 ± 0	MP	Pelagic
bombay duck	Harpodon nehereus	4.2 ± 0.73	MP	Demersal
red fish	Helicolenus dactylopterus	3.8 ± 0.6	MP	Demersal
scorpion fishes	Helicolenus hilgendorfi	3.2 ± 0.4	MP	Benthic
red Irish lord	Hemilepidotus hemilepidotus	3.5 ± 0.6	MP	Benthic
garfish	Hemiramphus robustus	3.3 ± 0.4	MP	Pelagic
cow sharks	Heptranchias perlo	4.2 ± 0.6	TP	Demersal
rock greenling	Hexagrammos lagocephalus	3.9 ± 0.5	MP	Demersal
sixgill shark	Hexanchus griseus	4.5 ± 0.2	TP	Demersal
honeycomb stingray	Himantura uarnak	3.6 ± 0.6	MP	Demersal
flathead flounder	Hippoglossoides dubius	3.6 ± 0.6	MP	Demersal
flathead sole	Hippoglossoides elassodon	3.6 ± 0.6	MP	Benthic
American plaice	Hippoglossoides platessoides	3.7 ± 0.5	MP	Benthic
**halibuts**	**Hippoglossus spp.**	**4.3 ± 0.65**	**MP**	**Benthic**
Pacific halibut	Hippoglossus stenolepis	4.1 ± 0.2	MP	Demersal
**squirrelfishes**	**Holocentrus spp.**	**3.5 ± 0.6**	**MP**	**Benthic**
cusk eel	Hoplobrotula armata	4 ± 0.66	MP	Benthic
orange roughy	Hoplostethus atlanticus	4.3 ± 0.1	TP	Demersal
beluga	Huso huso	4.1 ± 0.97	MP	Pelagic
ratfish	Hydrolagus barbouri	3.5 ± 0.37	MP	Demersal
greater sand eel	Hyperoplus lanceolatus	4.2 ± 0.7	MP	Demersal

walleye	Hyperprosopob argenteum	3.5 ± 0.5	MP	Demersal
shiner surf perch	Hyperprosopon ellipticum	3.4 ± 0.5	MP	Demersal
snowy grouper	Hyporthodus niveatus	4 ± 0.58	MP	Demersal
rainbow surf perch	Hypsurus caryi	3.3 ± 0.5	MP	Demersal
cheekspot goby	Ilypnus gilberti	3.2 ± 0.4	MP	Demersal
Japanese flathead	Inegocia japonica	3.7 ± 0.6	MP	Demersal
Indo-Pacific sailfish	Istiophorus platypterus	4.5 ± 0.3	TP	Pelagic
mako shark; shortfin mako	Isurus oxyrinchus	4.5 ± 0	TP	Pelagic
bigeye croaker	Johnius aneus	4 ± 0.69	TP	Benthic
Belanger's croaker	Johnius belangerii	3.3 ± 0.23	P	Benthic
karut croaker	Johnius carutta	4 ± 0.61	TP	Demersal
sin croaker	Johnius dussumieri	4.1 ± 0.7	TP	Demersal
sin croaker	Johnius sina	4.1 ± 0.7	TP	Demersal
soldier croaker	Johnius soldado	4 ± 0.61	TP	Demersal
stone flounder	Kareius bicoloratus	3.7 ± 0.6	MP	Demersal
skipjack tuna	Katsuwonus pelamis	3.8 ± 0.6	TP	Pelagic
gizzard shad	Konosirus punctatus	2.9 ± 0.22	H	Benthic
spiny flathead	Kumococius rodericensis	3.8 ± 0.6	MP	Demersal
bermuda sea chub	Kyphosus sectatrix	2 ± 0	H	Benthic
brassy chub	Kyphosus vaigiensis	2 ± 0	H	Benthic
hogfish	Lachnolaimus maximus	4.2 ± 0	MP	Demersal
longfin codling	Laemonema longipes	3.6 ± 0.4	MP	Demersal
pinfish	Lagodon rhomboides	4.4 ± 0	MP	Demersal
brokenline laternfish	Lampanyctus jordani	3.3 ± 0.4	MP	Pelagic
pinpoint lampfish	Lampanyctus regalis	3.2 ± 0.4	P	Pelagic
lamprey	Lampetra fluviatilis	4.5 ± 0.8	TP	Demersal
opah	Lampris guttatus	4.2 ± 0.62	TP	Pelagic
opah	Lamprius regius	4.2 ± 0.62	TP	Pelagic
deepsea fish	Lamprogrammus niger	3.7 ± 0.6	MP	Pelagic
yellow croakers	Larimichthys crocea	3.7 ± 0.56	MP	Demersal
sea perch; Japanese seabass	Lateolabrax japonicus	3.4 ± 0.43	MP	Demersal
striped trumpeter	Latris lineata	3.8 ± 0.6	MP	Pelagic
common ponyfish	Leiognathus equulus	3 ± 0.4	P	Demersal
long-finned slipmouth	Leiognathus fasciatus	3.3 ± 0.45	MP	Demersal
spot	Leiostomus xanthurus	3.9 ± 0.4	P	Benthic
Mediterranean codling	Lepidion lepidion	3.6 ± 0.6	MP	Benthic
escolar	Lepidocybium flavobrunneum	4.3 ± 0.67	TP	Pelagic
bay goby	Lepidogobius lepidus	3.3 ± 0.4	MP	Demersal
rock sole	Lepidopsetta bilineata	3.2 ± 0.4	MP	Benthic
Northern rock sole	Lepidopsetta polyxystra	3.3 ± 0.5	MP	Demersal
scabberfish	Lepidopus caudatus	3.9 ± 0.7	MP	Demersal
fourspot megrim	Lepidorhombus boscii	3.7 ± 0.6	MP	Benthic
megrim	Lepidorhombus whiffiagonis	4.2 ± 0.8	TP	Demersal
large-scaled gurnard	Lepidotrigla cavillone	3.2 ± 0.4	MP	Demersal
redbanded searobin	Lepidotrigla guentheri	3.5 ± 0.5	MP	Demersal
**gurnards**	**Lepidotrigla spp.**	**3.4 ±**	**MP**	**Demersal**
Pacific staghorn sculpin	Leptocottus armatus	3.7 ± 0.64	MP	Benthic
Savalai hairtail	Lepturacanthus savala	4.3 ± 0.76	TP	Pelagic

Pacific yellowtail emperor	Lethrinus atkinsoni	3.5 ± 0.5	MP	Demersal
grass emperor	Lethrinus laticaudis	4.5 ± 0.8	MP	Demersal
spangled emperor	Lethrinus nebulosus	3.3 ± 0.4	MP	Demersal
spotcheek emperor	Lethrinus rubrioperculatus	3.6 ± 0.6	MP	Demersal
sandy ray	Leucoraja circularis	3.5 ± 0.37	MP	Benthic
cuckoo ray	Leucoraja naevus	3.9 ± 0.6	MP	Benthic
dab	Limanda limanda	3.3 ± 0.4	MP	Demersal
marbled sole	Limanda yokohamae	3.5 ± 0.5	MP	Benthic
slender scorpionfish	Lioscorpius longiceps	3.2 ± 0.4	MP	Demersal
sand steenbras	Lithognathus mormyrus	3.4 ± 0.5	MP	Benthic
**mullet**	**Liza abua**	**2 ±**	**H**	**Benthic**
golden grey mullet	Liza aurata	2.5 ± 0.2	H	Benthic
**mullet**	**Liza dussumieri**	**2.7 ± 0.3**	**H**	**Benthic**
redeye mullet	Liza haematocheila	2.5 ± 0.19	H	Benthic
thicklip grey mullet	Liza ramada	2.6 ± 0.32	H	Benthic
**Indian shad**	**Ilisha indica**	**2 ± 0.1**	**H**	**Benthic**
tripletail	Lobotes surinamensis	4 ± 0.5	TP	Demersal
goosefish	Lophius americanus	4.5 ± 0.6	TP	Demersal
blackbellied angler	Lophius budegassa	4.5 ± 0.8	TP	Demersal
blackfin goosefish	Lophius gastrphysus	4.5 ± 0.6	TP	Demersal
sole	Lophius piscatorius	4.5 ± 0.8	TP	Demersal
	**Lophius spp.**	**4.5 ± 0.6**	**TP**	**Demersal**
tilefish	Lopholatilus chamaeleonticep	3.5 ± 0.4	MP	Demersal
rainwater killifish	Lucania parva	3.1 ± 0.4	P	Benthic
longsnout prickleback	Lumpenella longirostris	3.1 ± 0.3	MP	Demersal
mutton snapper	Lutjanus analis	3.9 ± 0.6	MP	Demersal
mangrove red snapper	Lutjanus argentimaculatus	4.5 ± 0.8	MP	Demersal
twospot red snapper	Lutjanus bohar	4.1 ± 0.7	TP	Demersal
blackfin snapper	Lutjanus buccanella	3.9 ± 0.57	MP	Demersal
Northern red snapper	Lutjanus campechanus	4 ± 0.59	TP	Demersal
Colorado snapper	Lutjanus colorado	3.1 ± 0	MP	Demersal
checkered snapper	Lutjanus decussatus	4 ± 0.67	TP	Pelagic
crimson snapper	Lutjanus erythropterus	4.5 ± 0.8	MP	Demersal
grey snapper	Lutjanus griseus	4.3 ± 0.8	TP	Demersal
dog snapper	Lutjanus jocu	4.4 ± 0.3	TP	Demersal
John's snapper	Lutjanus johnii	4.2 ± 0.7	TP	Demersal
common bluestripe snapper	Lutjanus kasmira	3.6 ± 0.6	MP	Demersal
mahogany snapper	Lutjanus mahogoni	4.3 ± 0.4	MP	Demersal
malabar red snapper	Lutjanus malabaricus	4.5 ± 0.8	TP	Demersal
onespot snapper	Lutjanus monostigma	4.3 ± 0.74	MP	Demersal
Southern red snapper	Lutjanus purpureus	3.6 ± 0.6	MP	Demersal
moses perch	Lutjanus russelli	4.3 ± 0.8	TP	Pelagic
humphead snapper	Lutjanus sanguineus	4.5 ± 0.8	MP	Pelagic
**snappers**	**Lutjanus spp.**	**4.5 ± 0.8**	**TP**	**Demersal**
lane snapper	Lutjanus synagris	3.8 ± 0.6	MP	Demersal
brownstripe red snapper	Lutjanus vitta	4.1 ± 0.7	TP	Demersal
eelpout	Lycodes hubbsi	3.5 ± 0.5	MP	Demersal
king weakfish	Macrodon ancylodon	3.9 ± 0.65	MP	Demersal

longnose snipe fish	Macroramphosus scolopax	3.5 ± 0.4	MP	Demersal
roughhead grenadier	Macrourus berglax	4.5 ± 0.8	MP	Benthic
blotched picarel	Maena maena	4.2 ± 0.7	TP	Demersal
**picarels**	**Maena spp.**	**4.2 ± 0.7**	**TP**	**Demersal**
blue marlin	Makaira nigricans	4.5 ± 0.3	TP	Pelagic
darkfin sculpin	Malacocottus zonurus	3.2 ± 0.4	MP	Demersal
splendid seabass	Malakichthys elegans	3.7 ± 0.5	MP	Demersal
lantern bellies	Malakichthys griseus	3.2 ± 0.4	P	Pelagic
silverbelly seaperch	Malakichthys wakiyae	4 ± 0.66	MP	Pelagic
capelin	Mallotus villosus	3.2 ± 0.3	P	Demersal
torpedo scad	Megalapsis cordyla	4.4 ± 0.8	TP	Pelagic
tarpon	Megalops atlanticus	4.5 ± 0	TP	Pelagic
haddock	Melanogrammus aeglefinus	4.1 ± 0.7	MP	Benthic
silverside	Menidia audens	3.1 ± 0.03	P	Demersal
Atlantic silversides	Menidia menidia	3.2 ± 0.3	P	Pelagic
Southern kingcroaker	Menticirrhus americanus	3.5 ± 0	MP	Demersal
Gulf kingcroaker	Menticirrhus littoralis	3.9 ± 0.6	MP	Benthic
Northern kingfish	Menticirrhus saxatilis	3.6 ± 0.5	MP	Benthic
kingfish	Menticirrhus undulatus	3.3 ± 0.5	MP	Demersal
whiting	Merlangius merlangus	4.4 ± 0.8	MP	Demersal
silver hake	Merluccius bilinearis	4.3 ± 0.7	MP	Demersal
hake	Merluccius capensis	4.5 ± 0.8	TP	Demersal
Aargentine hake	Merluccius hubbsi	4.2 ± 0.67	TP	Pelagic
European hake	Merluccius merluccius	4.4 ± 0.8	MP	Demersal
Pacific hake	Merluccius productus	4.4 ± 0.8	MP	Demersal
halfmoon	Microcanthus strigatus	3 ± 0.35	P	Demersal
blue whiting	Micromesistius poutassou	4 ± 0.7	MP	Demersal
whitemouth croaker	Micropogonias furnieri	3.3 ± 0.5	MP	Demersal
croaker	Micropogonias undulatus	3.3 ± 0.4	P	Demersal
lemon sole	Microstomus kitt	3.3 ± 0.4	MP	Benthic
dover sole	Microstomus pacificus	3.2 ± 0.1	MP	Demersal
ocean sunfish	Mola mola	3.7 ± 0.47	MP	Pelagic
ling	Molva molva	4.2 ± 0.73	MP	Demersal
fanbellied leatherjacket	Monacanthus chinensis	2.6 ± 0.3	H	Benthic
butter bream	Monodactylus argenteus	3 ± 0.33	P	Demersal
humpnose bigeye bream	Monotaxis grandoculis	3.4 ± 0	MP	Benthic
common mora	Mora moro	3.8 ± 0.55	MP	Demersal
white perch	Morone americana	3 ± 0.2	P	Demersal
striped bass	Morone saxatilis	4.5 ± 0.8	MP	Demersal
golden grey mullet	Mugil auratus	2.5 ± 0.2	H	Benthic
mullet	Mugil cephalus	2.1 ± 0.2	H	Benthic
white mullet	Mugil curema	2 ± 0	H	Benthic
mullet	Mugil gyrans	2 ± 0	H	Benthic
lebranche mullet	Mugil liza	2 ± 0	H	Benthic
mullet	Mugil platanus	2 ± 0	H	Benthic
mullet	Mugil soiuy	2.5 ± 0.19	H	Benthic
**mullets**	**Mugilidae spp.**	**2.5 ± 0.5**	**H**	**Benthic**
ureogenic goby	Mugilogobius abei	3.2 ± 0.5	P	Demersal

red goatfish	Mulloidichthys vanicolensis	3.6 ± 0.5	MP	Benthic
red mullet	Mullus barbatus	3.2 ± 0.4	P	Benthic
**mullets**	**Mullus spp.**	**3.2 ± 0.4**	**P**	**Benthic**
red mullet	Mullus surmuletus	3.4 ± 0.5	P	Benthic
Mediterranean moray	Muraena helena	4.2 ± 61	TP	Demersal
**morey eels**	**Muraena spp.**	**3.9 ± 0.6**	**MP**	**Demersal**
daggertooth pike conger	Muraenesox cinereus	4.1 ± 0.66	TP	Demersal
dogfish; gummy shark	Mustelus antarcticus	4.5 ± 0.59	TP	Demersal
dusky smoothhound	Mustelus canis	3.6 ± 0.2	MP	Demersal
dog shark	Mustelus griseus	3.5 ± 0.6	MP	Benthic
brown smoothhound shark	Mustelus henlei	3.6 ± 0.5	MP	Benthic
smalleye smoothhound	Mustelus higmani	3.6 ± 0.3	MP	Demersal
narrowfin smoothhound	Mustelus norrisi	3.9 ± 0.67	MP	Demersal
narrownose smoothhound	Mustelus schmitti	3.6 ± 0.5	MP	Demersal
black grouper	Mycteroperca bonaci	4.3 ± 0.5	TP	Demersal
gag	Mycteroperca microlepis	3.7 ± 0.6	MP	Demersal
scamp	Mycteroperca phenax	4.5 ± 0.8	TP	Demersal
	**Myctophiformes spp.**	**3.2 ± 0.4**	**P**	**Pelagic**
bright lanternfish	Myctophum phengodes	3.4 ± 0.5	MP	Pelagic
eagle ray	Myliobatis aquila	3.6 ± 0.54	MP	Benthic
bat eagle ray	Myliobatis californica	3.1 ± 0.3	MP	Benthic
bullnose eagle ray	Myliobatis freminvillei	3.4 ± 0	MP	Benthic
great sculpin	Myoxocephalus polyacanthoceph	4.1 ± 0.7	MP	Demersal
sculpin	Myoxocephalus quadricornis	3.7 ± 0.59	MP	Demersal
sculpin	Myoxocephalus scorpioides	3.4 ± 0.52	P	Demersal
shorthorn sculpin	Myoxocephalus scorpius	3.9 ± 0.4	MP	Demersal
giant electric ray	Narcine entemedor	3 ± 0.18	P	Benthic
whitemargin unicornfish	Naso annulatus	2.1 ± 0.09	H	Benthic
bluespine unicornfish	Naso unicornis	2.2 ± 0.11	H	Benthic
whitenose shark	Nasolamia velox	4.2 ± 0.74	TP	Pelagic
smalleye croaker	Nebris microps	3.6 ± 0.59	MP	Demersal
lemon shark	Negaprion brevirostris	4.3 ± 0.5	TP	Demersal
bony bream	Nematalosa come	2.8 ± 0.28	H	Pelagic
roosterfish	Nematistius pectoralis	4.5 ± 0.8	TP	Demersal
Japanese threadfin bream	Nemipterus japonicus	3.8 ± 0.51	MP	Demersal
notchedfin threadfin bream	Nemipterus peronii	3.7 ± 0.6	MP	Demersal
golden threadfin bream	Nemipterus virgatus	4 ± 0.57	MP	Demersal
catfish	Neoarius australis	3.3 ± 0.6	MP	Demersal
cusk eel	Neobythites sivicola	4 ± 0.66	MP	Demersal
round goby	Neogobius melanostomus	3.2 ± 0.4	P	Benthic
white sea catfish	Netuma barba	3.6 ± 0.4	MP	Demersal
giant catfish	Netuma thalassina	3.1 ± 0.3	P	Benthic
white flower croaker	Nibea albiflora	3.5 ± 0.37	MP	Demersal
soldier croaker	Nibea soldado	4 ± 0.61	TP	Demersal
**croakers**	**Nibea spp.**	**3.5 ±**	**MP**	**Demersal**
croaker	Nibea spp	4.5 ± 0.7	MP	Demersal
spiny eel	Notacanthus chemnitzii	3.5 ± 0.37	P	Benthic
Antarctic yellowbelly rockcod	Notothenia corriceps	2.8 ± 0.35	H	Benthic

marbled rockcod	Notothenia rossii	3.6 ± 0.6	MP	Demersal
saddled seabream	Oblada melanura	3 ± 0.1	P	Benthic
yellowtail snapper	Ocyurus chrysurus	4 ± 0.3	TP	Demersal
eelgoby	Odontamblyopus rubicundus	3.8 ± 0.5	MP	Benthic
sand tiger shark	Odontaspis taurus	4.5 ± 0.8	TP	Demersal
	Odontesthes argentinensis	3.3 ± 0.5	P	Pelagic
**silversides**	**Odontesthes spp.**	**3.3 ± 0.5**	**P**	**Pelagic**
red-toothed triggerfish	Odonus niger	3.2 ± 0.32	P	Benthic
castin leatherjacket	Oligoplites saliens	3.8 ± 0.62	MP	Pelagic
leatherjacket	Oligoplites saurus	4.3 ± 0.5	TP	Demersal
oyster blenny	Omobranchus anolius	2.7 ± 0.2	H	Benthic
salmon	Oncorhynchus gorbuscha	4.2 ± 0.7	MP	Demersal
chum salmon	Oncorhynchus keta	3.5 ± 0.5	MP	Demersal
coho	Oncorhynchus kisutch	4.2 ± 0.7	MP	Demersal
sockeye salmon	Oncorhynchus nerka	3.7 ± 0.4	MP	Demersal
chinook salmon	Oncorhynchus tshawytscha	4.4 ± 0.7	TP	Demersal
lingcod	Ophiodon elongatus	4.3 ± 0.72	TP	Demersal
Altantic thread herring	Opisthonema oglinum	4.5 ± 0	TP	Demersal
knifejaws	Oplegnathus fasciatus	3.6 ± 0.5	MP	Demersal
Gulf toadfish	Opsanus beta	3.7 ± 0.6	MP	Demersal
pigfish	Orthopristis chrysoptera	3.4 ± 0	MP	Demersal
corocoro gruunt	Orthopristis ruber	3.6 ± 0.2	MP	Benthic
tiger-toothed croaker	Otolithes ruber	3.6 ± 0.6	MP	Demersal
silvery croaker	Otolithus argenteus	3.6 ± 0.6	MP	Demersal
axillary bream	Pagellus acarne	3.5 ± 0.5	MP	Benthic
blackspot seabream	Pagellus bogaraveo	3.7 ± 0.56	MP	Demersal
common pandora	Pagellus erythrinus	3.4 ± 0.5	MP	Benthic
emerald rockcod	Pagothenia bernacchii	3.3 ± 0.4	MP	Demersal
striped rockcod	Pagothenia hansoni	3.9 ± 0.6	MP	Demersal
snapper	Pagrus auratus	3.3 ± 0.5	MP	Demersal
red seabream	Pagrus major	3.7 ± 0.49	MP	Benthic
red porgy	Pagrus pagrus	3.7 ± 0.6	MP	Demersal
silver pomfret	Pampus argenteus	3.1 ± 0.4	P	Demersal
chinese silver pomfret	Pampus chinensis	3.6 ± 0.38	MP	Pelagic
**pomfrets**	**Pampus spp.**	**3.1 ± 0.4**	**P**	**Demersal**
kelp bass	Paralabrax clathratus	3.9 ± 0.6	MP	Demersal
spotted sand bass	Paralabrax maculatofasciatus	4.2 ± 0.6	MP	Demersal
Gulf flounder	Paralichthys albigutta	4.2 ± 0.7	TP	Demersal
halibut	Paralichthys californicus	4.5 ± 0.63	MP	Benthic
summer flounder	Paralichthys dentatus	4.5 ± 0.6	MP	Benthic
flounder	Paralichthys lethostigma	3.6 ± 0.6	MP	Benthic
Japanese flounder	Paralichthys olivaceus	4.4 ± 0.8	TP	Demersal
	Paralichthys orbignyanus	3.5 ± 0.54	MP	Demersal
**flounders**	**Paralichthys spp.**	**4.4 ± 0.6**	**MP**	**Benthic**
speckled flounder	Paralichthys woolmani	4.5 ± 0.8	TP	Demersal
creolefish	Paranthias furcifer	3.2 ± 0.1	P	Demersal
black pomfret	Parastromateus niger	2.9 ± 0.35	P	Benthic
dash-and-dot goatfish	Parupeneus barberinus	3.2 ± 0.4	P	Demersal

yellow striped goatfish	Parupeneus chrysopleuron	3.5 ± 0.7	MP	Benthic
gold-saddle goatfish	Parupeneus cyclostomus	4.2 ± 0.7	MP	Demersal
manybar goatfish	Parupeneus multifasciatus	3.5 ± 0.6	MP	Demersal
bigeye croaker	Pennahia anea	4 ± 0.69	TP	Benthic
white croaker	Pennahia argentata	3.5 ± 0.43	MP	Demersal
American harvestfish	Peprilus paru	4.5 ± 0	TP	Pelagic
perch	Perca fluviatilis	4.4 ± 0.8	TP	Demersal
mudskipper	Periophtalmodon schlosseri	2.5 ± 0.5	P	Demersal
armored gurnard	Peristedion cataphractum	3.6 ± 0.4	MP	Benthic
oyster blenny	Petroscirtes anolius	2.7 ± 0.2	H	Benthic
white surf perch	Phanerodon furcatus	3.4 ± 0.5	MP	Demersal
forkbeard	Phycis blennoides	3.7 ± 0.66	MP	Demersal
forkbeard	Phycis phycis	4.3 ± 0.3	MP	Demersal
glass eel	Pisodonophis boro	4 ± 0.71	TP	Demersal
flounder	Platichthys flesus	3.2 ± 0.4	MP	Benthic
sand flathead	Platycephalus bassensis	4.3 ± 0.75	TP	Demersal
flathead	Platycephalus fuscus	4.1 ± 0.52	MP	Demersal
bartail flathead	Platycephalus indicus	3.6 ± 0.6	MP	Demersal
spiny flathead	Platycephalus rodericensis	3.8 ± 0.6	MP	Demersal
two-striped sweetlips	Plectorhinchus albovittatus	4 ± 0.66	MP	Demersal
harlequin sweetlips	Plectorhinchus chaetodonoides	3.8 ± 0.6	MP	Demersal
yellowband sweetlip	Plectorhinchus diagrammus	3.4 ± 0.5	MP	Benthic
lemon sweetlips	Plectorhinchus flavomaculatus	4 ± 0.66	MP	Demersal
blackall	Plectorhinchus pictus	3.5 ± 0.5	MP	Demersal
sweetlips	Plectorhynchus cinctus	3.5 ± 0.49	MP	Demersal
leopard coral grouper	Plectropomus leopardus	4.7 ± 0.7	TP	Demersal
silverfish	Pleuragramma antarcticum	3 ± 0	P	Pelagic
Atka mackerel	Pleurogrammus monopterygius	3.3 ± 0.4	MP	Demersal
plaice	Pleuronectes platessa	3.3 ± 0.4	MP	Benthic
english sole	Pleuronectes vetulus	3.4 ± 0.46	MP	Demersal
**flounders**	**Pleuronichthys spp.**	**3.3 ± 0.4**	**MP**	**Benthic**
striped eel catfish	Plotosus anguillaris	3.5 ± 0.6	MP	Benthic
striped eel catfish	Plotosus lineatus	3.5 ± 0.6	MP	Benthic
drum	Pogonias cromis	3.9 ± 0.62	MP	Demersal
black pollock	Pollachius pollachius	4.3 ± 0.3	TP	Demersal
saithe	Pollachius virens	4.4 ± 0.8	MP	Demersal
six-threads threadfin	Polydactylus sextarius	3.8 ± 0.57	MP	Benthic
dragon fishes	Polyipnus spinifer	3.3 ± 0.4	MP	Demersal
paradise threadfin	Polynemus paradiseus	3.9 ± 0.6	MP	Demersal
wreckfish	Polyprion americanus	4.1 ± 0.64	TP	Demersal
Hapuku wreckfish	Polyprion oxygeneios	4.5 ± 0.77	TP	Pelagic
silver grunt	Pomadasys argenteus	3.4 ± 0.5	MP	Demersal
sand grunt	Pomadasys branickii	3.4 ± 0.5	MP	Demersal
silver grunt	Pomadasys hasta	3.4 ± 0.5	MP	Demersal
raucous grunt	Pomadasys leuciscus	3.2 ± 0.46	MP	Demersal
bluefish; tailor	Pomatomus saltator; saltatrix	4.5 ± 0.6	TP	Demersal
common goby	Pomatoschistus microps	3.3 ± 0.4	P	Demersal
sand goby	Pomatoschistus minutus	3.2 ± 0.4	P	Demersal

Atlantic bigeye	Priacanthus arenatus	4 ± 0	MP	Demersal
moontail bullseye	Priacanthus hamrur	3.6 ± 0.5	MP	Demersal
blue shark	Prionace glauca	4.2 ± 0.7	TP	Pelagic
sharp-tooth snapper	Pristimoides typus	4.2 ± 0.61	TP	Benthic
smalltooth sawfish	Pristis pectinata	4.5 ± 0.8	TP	Demersal
pompano	Psenopsis anomala	4 ± 0.3	MP	Demersal
turbot	Psetta maxima	4 ± 0.63	MP	Benthic
Indian halibut	Psettodes erumei	4.4 ± 0.8	TP	Demersal
gwelly; white trevally	Pseudocaranx dentex	3.9 ± 0.6	MP	Benthic
winter flounder	Pseudopleuronectes americanus	2.8 ± 0.4	MP	Benthic
marbled sole	Pseudopleuronectes yokohamae	3.5 ± 0.5	MP	Demersal
Japanese flounder	Pseudorhombus javanicus	3.5 ± 0.37	MP	Benthic
flounder	Pseudorhombus jenynsii	3.5 ± 0.37	MP	Benthic
Malayan flounder	Pseudorhombus malayanus	3.5 ± 0.37	MP	Benthic
large yellow croaker	Pseudosciaena crocea	3.7 ± 0.56	MP	Demersal
small yellow croaker	Pseudosciaena polyactis	3.6 ± 0.63	MP	Demersal
Japanese gissu	Pterothrissus gissu	3.3 ± 0.5	MP	Demersal
cobia	Rachycentron canadum	4 ± 0	TP	Pelagic
cobia	Rachycentron canadus	4 ± 0.6	TP	Demersal
starry ray	Raja asterias	3.5 ± 0.37	MP	Benthic
thornback ray	Raja clavata	3.8 ± 0.2	MP	Demersal
shagreen ray	Raja fullonica	3.5 ± 0.37	MP	Benthic
round ray	Raja fyllae	3.3 ± 0.5	MP	Benthic
skate	Raja kenojei	4.1 ± 0.74	TP	Benthic
skates	Raja kwangtungensis	3.8 ± 0.6	MP	Demersal
winter skate	Raja miraletus	3.8 ± 0.74	MP	Benthic
long nose skate	Raja oxyrhynchus	3.5 ± 0.37	MP	Benthic
starry ray	Raja radiata	4.2 ± 0.3	TP	Benthic
**rays; skates**	**Raja spp.**	**3.8 ± 0**	**MP**	**Benthic**
skate	Raja texana	3.8 ± 0.58	MP	Benthic
short mackerel	Rastrelliger brachysoma	2.7 ± 0.31	H	Benthic
Indian mackerel	Rastrelliger kanagurta	3.2 ± 0.38	MP	Pelagic
short mackerel	Rastrelliger neglectus	2.7 ± 0.31	H	Benthic
halibut	Reinhardtius hippoglossoides	4.5 ± 0.8	TP	Demersal
speckled guitarfish	Rhinobatos glaucostigma	3.5 ± 0.6	MP	Benthic
shovelnose guitarfish	Rhinobatos productus	3.6 ± 0.6	MP	Benthic
cownose ray	Rhinoptera bonasus	3.2 ± 0	MP	Benthic
Pacific cownose ray	Rhinoptera steindachnerii	3.6 ± 0.5	MP	Benthic
Pacific sharpnose shark	Rhizoprionodon longurio	4.2 ± 0.7	TP	Demersal
Atlantic sharpnose shark	Rhizoprionodon terraenovae	4.3 ± 0.8	TP	Pelagic
vermillion snapper	Rhomboplites aurorubens	4.3 ± 0.6	TP	Demersal
striped bass	Roccus saxatilis	4.5 ± 0.8	MP	Demersal
spotfin croaker	Roncador stearasii	3.3 ± 0.44	MP	Benthic
**slickhead fishes**	**Rouleina spp.**	**3.3 ± 0.4**	**MP**	**Benthic**
Norwegian salmon	Salmo salar	4.4 ± 0.1	MP	Demersal
**salmons**	**Salmo spp.**	**4 ± 0**	**MP**	**Demersal**
sea trout	Salmo trutta	3.2 ± 0.4	MP	Demersal
Arctic char	Salvelinus alpinus	4.3 ± 0.8	MP	Demersal

dolly varden	Salvelinus malma	4.2 ± 0.7	MP	Demersal
Atlantic bonito	Sarda sarda	4.5 ± 0.7	TP	Pelagic
sardine; European pilchard	Sardina pilchardus	3.1 ± 0.2	P	Pelagic
anchovy	Sardinella anchovia	3.4 ± 0.5	MP	Pelagic
guilt sardine	Sardinella aurita	3.4 ± 0.5	MP	Pelagic
Brazilian sardinella	Sardinella brasiliensis	3.1 ± 0.3	P	Pelagic
sind sardinella	Sardinella sindensis	2.9 ± 0.3	P	Pelagic
Pacific sardine	Sardinops sagax	2.4 ± 0.1	P	Pelagic
salema	Sarpa salpa	2 ± 0	H	Benthic
gracile lizardfish	Saurida gracilis	4.2 ± 0.7	MP	Benthic
clouded lizardfish	Saurida nebulosa	4.2 ± 0.73	MP	Demersal
greater lizardfish	Saurida tumbil	4.4 ± 0.8	TP	Demersal
brushtooth lizardfish	Saurida undosquamis	4.5 ± 0.8	TP	Demersal
blue-barred parrotfish	Scarus ghobban	2 ± 0	H	Benthic
blunt-head parrotfish	Scarus microrhinos	2 ± 0.01	H	Benthic
rivulated parrotfish	Scarus rivulatus	2 ± 0	H	Benthic
yellowband parrotfish	Scarus schlegeli	2 ± 0	H	Benthic
daisy parrotfish	Scarus sordidus	2 ± 0	H	Benthic
**parrotfishes**	**Scarus spp.**	**2 ± 0**	**H**	**Benthic**
Imperial blackfish	Schedophilus ovalis	3.5 ± 0.57	MP	Pelagic
goatee croaker	Sciaena russelli	3.5 ± 0.5	MP	Demersal
red drum	Sciaenops ocellatus	4.1 ± 0.7	MP	Demersal
spadenose shark	Scoliodon laticaudus	3.8 ± 0.6	MP	Pelagic
Pacific/chub mackerel	Scomber japonicus	3.1 ± 0.4	MP	Pelagic
mackerel	Scomber scombrus	3.7 ± 0.6	MP	Pelagic
talang queenfish	Scomberoides commersonianus	4.5 ± 0.8	TP	Pelagic
doublespotted queenfish	Scomberoides lysan	4.5 ± 0.8	TP	Pelagic
king mackerel	Scomberomorus cavalla	4.5 ± 0.8	TP	Pelagic
narrowbarred spanish mackerel	Scomberomorus commerson	4.5 ± 0.8	TP	Pelagic
Indo-Pacific king mackerel	Scomberomorus guttatus	4.3 ± 0.67	TP	Pelagic
Korean mackerel; Korean seerfish	Scomberomorus koreanus	4.2 ± 0.74	TP	Pelagic
Altantic spanish mackerel	Scomberomorus maculatus	4.5 ± 0.5	TP	Pelagic
cero	Scomberomorus regalis	4.5 ± 0.4	MP	Pelagic
Pacific sierra	Scomberomorus sierra	4.5 ± 0.8	TP	Pelagic
**mackerels**	**Scomberomorus spp.**	**4.4 ± 0.7**	**MP**	**Pelagic**
Japanese spanish mackerel	Scomberomrus niphonius	4.5 ± 0.8	TP	Pelagic
**windowpane**	**Scophthalmus aquosus**	**3 ± 0**	**MP**	**Demersal**
brill	Scophthalmus rhombus	4.4 ± 0.1	MP	Benthic
black scorpionfish	Scorpaena porcus	3.9 ± 0.7	MP	Demersal
**scorpionfishes**	**Scorpaena spp.**	**3.9 ± 0.7**	**MP**	**Demersal**
lesser spotted dogfish	Scyliorhinus canicula	3.7 ± 0.6	MP	Demersal
Pacific ocean perch	Sebastes alutus	3.5 ± 0.3	MP	Demersal
kelp rockfish	Sebastes atrovirens	3.4 ± 0.48	MP	Demersal
red fish	Sebastes marinus	4 ± 0.68	MP	Pelagic
black rockfish	Sebastes melanops	4.4 ± 0.8	MP	Demersal
canary rockfish	Sebastes pinniger	3.8 ± 0.61	MP	Demersal
scorpionfish	Sebastiscus marmoratus	3.6 ± 0.6	MP	Demersal
broadbanded thornyhead	Sebastolobus macrochir	3.3 ± 0.5	MP	Demersal

oxeye scad	Selar boops	3.5 ± 0.41	MP	Demersal
bigeye scad	Selar crumenophthalmus	4.1 ± 0.7	TP	Pelagic
yellowstripe scad	Selaroides leptolepis	3.5 ± 0.5	MP	Demersal
Atlantic moonfish	Selene setapinnis	3.7 ± 0.52	MP	Demersal
lookdown	Selene vomer	4.3 ± 0.6	MP	Demersal
striped butterfish	Selenotoca multifasciata	2.9 ± 0.3	H	Benthic
amberjack	Seriola dumerili	4.5 ± 0.8	TP	Pelagic
yellowtail	Seriola lalandi	4.1 ± 0.3	TP	Pelagic
almaco jack	Seriola rivoliana	4.5 ± 0.8	TP	Pelagic
banded rudderfish	Seriola zonata	4.5 ± 0.8	TP	Demersal
comber	Serranus cabrilla	3.4 ± 0.3	MP	Demersal
brown comber	Serranus hepatus	3.5 ± 0.4	MP	Demersal
barred rockcod	Serranus novemcinctus	3.7 ± 0.56	MP	Demersal
painted comber	Serranus scriba	3.8 ± 0.6	MP	Demersal
obtuse barracuda	Shyraena obstusata	4.5 ± 0.8	TP	Pelagic
slimy spinefoot	Siganus canaliculatus	2.8 ± 0.31	H	Benthic
houttuyn	Siganus fuscescens	2.3 ± 0.13	H	Benthic
streaked spinefoot	Siganus javus	2.4 ± 0.8	H	Benthic
rabbit fish	Siganus oramin	2.8 ± 0.31	H	Benthic
gold-spotted spinefoot	Siganus punctatus	2.8 ± 0.1	H	Benthic
little spinefoot	Siganus spinus	2 ± 0	H	Benthic
rabbitfish	Siganus vermiculatus	2 ± 0	H	Benthic
summer whiting	Sillago ciliata	3.2 ± 0.4	P	Benthic
silver sillago	Sillago sihama	3.4 ± 0.5	P	Demersal
snubnosed eel	Simenchelys parasitica	3.7 ± 0.54	MP	Demersal
sole	Solea solea	3.1 ± 0.3	P	Benthic
frogfish	Solea vulgaris	3.1 ± 0.3	P	Benthic
Greenland shark	Somniosus microcephalus	4.2 ± 0.6	TP	Demersal
seabream	Sparus aurata	3.8 ± 0.6	MP	Demersal
yellowfin porgy	Sparus latus	3.2 ± 0.5	P	Benthic
bullseye puffer	Sphoeroides annulatus	3.1 ± 0.44	MP	Demersal
great barracuda	Sphyraena barracuda	4.5 ± 0.6	TP	Pelagic
bigeye barracuda	Sphyraena forsteri	4.3 ± 0.8	TP	Pelagic
Guachanche barracuda	Sphyraena guachancho	3.9 ± 0.6	MP	Pelagic
Japanese barracuda	Sphyraena japonica	4.2 ± 0.73	MP	Pelagic
pickhandle barracuda	Sphyraena jello	4.5 ± 0.8	TP	Pelagic
obtuse barracuda	Sphyraena obtusata	4.5 ± 0.8	TP	Pelagic
**barracudas**	**Sphyraena spp.**	**4.3 ± 0.8**	**TP**	**Pelagic**
barracuda	Sphyraena sphyraena	4 ± 0.51	TP	Pelagic
scalloped hammerhead	Sphyrna lewini	4.1 ± 0.5	TP	Pelagic
bonnethead shark	Sphyrna tiburo	3.9 ± 0.5	TP	Demersal
hammerhead shark	Sphyrna zygaena	4.5 ± 0.8	TP	Pelagic
blotched picarel	Spicara maena	4.2 ± 0.7	TP	Demersal
picarel	Spicara smaris	3 ± 0	P	Demersal
sprat	Sprattus sprattus	3 ± 0	P	Pelagic
spiny dogfish	Squalus acanthias	4.3 ± 0.7	TP	Demersal
longnose spurdog	Squalus blainvillei	4 ± 0.6	TP	Pelagic
dogfish shark	Squalus japonicus	4.4 ± 0.7	TP	Demersal

shortnose spurdog	Squalus megalops	4.3 ± 0.3	TP	Demersal
shortspine spurdog	Squalus mitsukurii	4.4 ± 0.3	TP	Demersal
Argentine angelshark	Squatina argentina	4.1 ± 0.7	MP	Benthic
Northern lampfish	Stenobrachius leucopsarus	3.2 ± 0.3	P	Demersal
garnet lanternfish	Stenobrachius nannochir	3.1 ± 0.2	P	Demersal
scup; porgy	Stenotomus chrysops	3.8 ± 0.1	MP	Benthic
Chinese silver pomfret	Stromateus sinensis	3.6 ± 0.38	MP	Pelagic
halfmoon triggerfish	Sufflamen chrysoptera	3.5 ± 0.41	MP	Demersal
**lanternfishes**	**Symbolophorus spp.**	**3.4 ± 0.5**	**MP**	**Pelagic**
tongue sole	Symphurus nigrescens	3.3 ± 0.4	MP	Demersal
Japanese splitfin	Synagrops japonicus	4.3 ± 0.73	MP	Demersal
shortdorsal cutthroat eel	Synaphobranchus brevidorsalis	4.1 ± 0.6	MP	Benthic
Kaup’s arrowtooth eel	Synaphobranchus kaupii	4.1 ± 0.6	TP	Demersal
goby	Synechogobius hasta	3 ± 0	P	Demersal
inshore lizardfish	Synodus foetens	4.5 ± 0	TP	Demersal
lizardfish	Synodus macrops	4 ± 0.67	MP	Demersal
sickle pomfret	Taractichthys steindachneri	4.3 ± 0.6	MP	Pelagic
tautog	Tautoga onitis	3.3 ± 0.47	MP	Demersal
cunner	Tautogolabrus adspersus	3.7 ± 0.2	MP	Benthic
Indian shad	Tenualosa ilisha	2 ± 0.1	H	Benthic
Altantic white marlin	Tetrapturus albidus	4.5 ± 0.4	TP	Pelagic
spearfish	Tetrapturus angustirostris	4.5 ± 0.76	TP	Pelagic
striped marlin	Tetrapturus audax	4.6 ± 0.8	TP	Pelagic
file fish	Thamnaconus hypargyreus	3.4 ± 0.44	P	Demersal
**bluefin leatherjacket**	**Thamnaconus septentrionalis**	**3.4 ± 0.4**	**MP**	**Benthic**
pollock	Theragra chalcogramma	3.5 ± 0.5	P	Demersal
small-scale banded therapon	Therapon puta	3.1 ± 0.41	P	Demersal
China anchovy	Thrissa kammalensis	3.1 ± 0.36	P	Pelagic
oblique-jaw thryssa	Thryssa purava	3.5 ± 0.5	MP	Pelagic
albacore tuna	Thunnus alalunga	4.3 ± 0.7	TP	Pelagic
yellowfin tuna	Thunnus albacares	4.3 ± 0.7	TP	Pelagic
blackfin tuna	Thunnus atlanticus	4.4 ± 0.3	TP	Pelagic
bluefin tuna	Thunnus maccoyii	4.2 ± 0.6	TP	Pelagic
bigeye tuna	Thunnus obesus	4.5 ± 0	TP	Pelagic
Pacific bluefin tuna	Thunnus orientalis	4.2 ± 0.6	TP	Pelagic
**tunas**	**Thunnus spp.**	**4.2 ± 0.6**	**TP**	**Pelagic**
Atlantic bluefin tuna	Thunnus thynnus	4.4 ± 0.8	TP	Pelagic
long tail tuna	Thunnus tonggol	4.5 ± 0.8	TP	Pelagic
snoek	Thyrsites atun	3.7 ± 0.6	MP	Pelagic
electric ray	Torpedo nobiliana	4.5 ± 0.6	TP	Demersal
snubnose pompano	Trachinotus blochii	3.7 ± 0.46	MP	Benthic
pompano	Trachinotus carolinus	3.5 ± 0.6	MP	Benthic
permit	Trachinotus falcatus	4 ± 0.2	MP	Benthic
paloma pompano	Trachinotus paitensis	3.7 ± 0.55	MP	Benthic
greater weever	Trachinus draco	4.2 ± 0.71	TP	Demersal
horse mackerel	Trachurus japonicus	3.4 ± 0.45	MP	Pelagic
horse mackerel	Trachurus mediterraneus	3.6 ± 0.4	MP	Pelagic
blue jack mackerel	Trachurus picturatus	3.3 ± 0.42	MP	Demersal

Atlantic horse mackerel	Trachurus trachurus	3.6 ± 0.6	MP	Pelagic
sharp-spined notothenia	Trematomus centronotus	3.3 ± 0.4	MP	Demersal
blunt scalyhead	Trematomus eulepidotus	3.3 ± 0.5	MP	Pelagic
crocodile icefish	Trematomus newnes	3.7 ± 0.6	MP	Demersal
leopard shark	Triakis semifasciata	3.7 ± 0.5	TP	Benthic
large-headed cutlassfish	Trichiurus haumela	4.5 ± 0.8	TP	Pelagic
largehead hairtail	Trichiurus japonicus	4.5 ± 0.8	MP	Pelagic
largehead hairtail	Trichiurus lepturus	4.5 ± 0.8	MP	Pelagic
Shimofuri goby	Tridentiger bifasciatus	3.4 ± 0.4	MP	Demersal
gurnard	Trigla lucerna	3.7 ± 0.6	MP	Demersal
piper	Trigla lyra	3.5 ± 0.5	MP	Demersal
streaked gurnard	Trigloporus lastoviza	3.4 ± 0.5	MP	Demersal
Norway pout	Trisopterus esmarkii	3.2 ± 0.4	MP	Pelagic
bib	Trisopterus luscus	3.7 ± 0.6	MP	Benthic
poor cod	Trisopterus minutus	3.8 ± 0.5	MP	Benthic
longtom	Tylosurus gavialoides	4.4 ± 0.8	TP	Benthic
sand drum	Umbrina coroides	3.1 ± 0.2	P	Demersal
goat fish	Upeneus japonicus	3.6 ± 0.38	MP	Demersal
goldband goatfish	Upeneus moluccensis	3.6 ± 0.6	MP	Pelagic
Brazilian codling	Urophycis brasiliensis	3.8 ± 0.6	MP	Demersal
white hake	Urophycis tenuis	4.2 ± 0.7	MP	Demersal
kanda	Valamugil engeli	2.5 ± 0.2	P	Demersal
bluespot grey mullet	Valamugil seheli	2.3 ± 0.14	H	Benthic
swordfish	Xiphias gladius	4.5 ± 0.6	TP	Pelagic
banded guitarfish	Zapteryx exasperata	3.6 ± 0.53	MP	Benthic
halfbeak	Zenarchopterus buffonis	2.5 ± 0.5	P	Pelagic
deep-sea fish	Zenion hololepis	3.7 ± 0.6	MP	Demersal
dories	Zenopsis nebulosa	4 ± 0.65	MP	Benthic
john dory	Zeus faber	4.5 ± 0.8	TP	Demersal
eelpout	Zoarces viviparus	3.5 ± 0.49	MP	Demersal
grass goby	Zosterisessor ophiocephalus	3.1 ± 0.4	MP	Benthic

Note: Bold selections reflect estimated trophic and ecological information (FishBase)

Supplemental Table 8. Seafood Database Reference List

Adams D.H. (2004). Total mercury levels in tunas from offshore waters of the Florida Atlantic coast. *Mar Pollut Bull*, 49, 659-663.

Adams D.H. & McMichael R.H. (1999). Mercury levels in four species of sharks from the Atlantic coast of Florida. *Fish B-Noaa*, 97, 372-379.

Adams D.H. & McMichael R.H. (2007). Mercury in king mackerel, *Scomberomorus cavalla*, and Spanish mackerel, *S. maculatus*, from waters of the south-eastern USA: regional and historical trends. *Mar Freshwater Res*, 58, 187-193.

Adams D.H., McMichael R.H. & Henderson G.E. (2003). Florida Marine Research Institute Technical Reports: Mercury levels in marine and estuarine fishes of Florida1989-2001. In. Florida Fish and Wildlife Conservation Commission, Florida Marine Research Institution.

Adams D.H., Sonne C., Basu N., Dietz R., Nam D.H., Leifsson P.S. & Jensen A.L. (2010). Mercury contamination in spotted seatrout, *Cynoscion nebulosus*: An assessment of liver, kidney, blood, and nervous system health. *Sci Total Environ*, 408, 5808-5816.

Adeyemi D., Ukpo G., Anyakora C. & Uyimadu J. (2009). Polychlorinated biphenyl in fish samples from Lagos Lagoon, Nigeria. *African Journal of Biotechnology*, 8, 2811-2815.

Afonso C., Lourenco H.M., Pereira C., Martins M.F., Carvalho M.L., Castro M. & Nunes M.L. (2008). Total and organic mercury, selenium and alpha-tocopherol in some deep-water fish species. *J Sci Food Agr*, 88, 2543-2550.

Al-Shwafi N., Al-trabeen K. & Rasheed M. (2009). Organochlorine pesticides and polychlorinated biphenyls carcinogens residual in some fish and shell fish of Yemen. *Jordan Journal of Biological Sciences*, 2, 23-28.

Andersen J.L. & Depledge M.H. (1997). A survey of total mercury and methylmercury in edible fish and invertebrates from Azorean waters. *Marine Environmental Research*, 44, 331-350.

Andersson O., Linder C.E., Olsson M., Reutergardh L., Uvemo U.B. & Wideqvist U. (1988). Spatial differences and temporal trends of organochlorine compounds in biota from the northwestern hemisphere. *Archives of Environmental Contamination and Toxicology*, 17, 755-765.

Arcos J.M., Ruiz X., Bearhop S. & Furness R.W. (2002). Mercury levels in seabirds and their fish prey at the Ebro Delta (NW Mediterranean): the role of trawler discards as a source of contamination. *Mar Ecol Prog Ser*, 232, 281-290.

Atuma S., Linder C.-E., Bergh A., Wicklund-Glynn A., Andersson O. & Jahnsson H. (1996). Levels of selected coplanar PCBs in fish from the Swedish water environment. *Organohalogen Compounds*, 28, 4.

Baeyens W., Leermakers M., Papina T., Saprykin A., Brion N., Noyen J., De Gieter M., Elskens M. & Goeyens L. (2003). Bioconcentration and biomagnification of mercury and methylmercury in North Sea and Scheldt estuary fish. *Arch Environ Contam Toxicol*, 45, 498-508.

Bank M.S., Chesney E., Shine J.P., Maage A. & Senn D.B. (2007). Mercury bioaccumulation and trophic transfer in sympatric snapper species from the Gulf of Mexico. *Ecol Appl*, 17, 2100-2110.

Baptista J., Pato P., Pereira E., Duarte A.C. & Pardal M.A. (2013). PCBs in the fish assemblage of a southern European estuary. *Journal of Sea Research*, 76, 22-30.

Baron E., Rudolph I., Chiang G., Barra R., Eljarrat E. & Barcelo D. (2013). Occurrence and behavior of natural and anthropogenic (emerging and historical) halogenated compounds in marine biota from the Coast of Concepcion (Chile). *Sci Total Environ*, 461, 258-264.

Barska I. & Skrzynski I. (2003). Contents of methylmercury and total mercury in Baltic Sea fish and fish products. In. Bulletin of the Sea Fisheries Institute, pp. 3-15.
Basturk O., Dogan M., Salihoglu I. & Balkas T.I. (1980). DDT, DDE, and PCB residues in fish, crustaceans and sediments from the eastern Mediterranean coast of Turkey. *Mar Pollut Bull*, 11, 191-195.
Bayarri S., Baldassarri L.T., Iacovella N., Ferrara F. & di Domenico A. (2001). PCDDs, PCDFs, PCBs and DDE in edible marine species from the Adriatic Sea. *Chemosphere*, 43, 601-610.
Bayen S., Wurl O., Karuppiah S., Sivasothi N., Lee H.K. & Obbard J.P. (2005). Persistent organic pollutants in mangrove food webs in Singapore. *Chemosphere*, 61, 303-313.
Bebianno M.J., Santos C., Canario J., Gouveia N., Sena-Carvalho D. & Vale C. (2007). Hg and metallothionein-like proteins in the black scabbardfish *Aphanopus carbo*. *Food Chem Toxicol*, 45, 1443-1452.
Bloom N.S. (1992). On the chemical form of mercury in edible fish and marine invertebrate tissue. *Canadian Journal of Fisheries and Aquatic Sciences*, 49, 1010-1017.
Bodin N., Abarnou A., Fraisse D., Defour S., Loizeau V., Le Guellec A.M. & Philippon X. (2007). PCB, PCDD/F and PBDE levels and profiles in crustaceans from the coastal waters of Brittany and Normandy (France). *Mar Pollut Bull*, 54, 657-668.
Boon J.P., Lewis W.E., Tjoen-A-Choy M.R., Allchin C.R., Law R.J., de Boer J., ten Hallers-Tjabbes C.C. & Zegers B.N. (2002). Levels of polybrominated diphenyl ether (PBDE) flame retardants in animals representing different trophic levels of the North Sea food web. *Environ Sci Technol*, 36, 4025-4032.
Borghesi N., Corsolini S., Leonards P., Brandsma S., de Boer J. & Focardi S. (2009). Polybrominated diphenyl ether contamination levels in fish from the Antarctic and the Mediterranean Sea. *Chemosphere*, 77, 693-698.
Braune B.M. (1987). Mercury accumulation in relation to size and age of Atlantic herring (*Clupea harengus harengus*) from the southwestern Bay of Fundy, Canada. *Archives of Environmental Contamination and Toxicology*, 16, 311-320.
Bright D.A., Dushenko W.T., Grundy S.L. & Reimer K.J. (1995). Effects of local and distant contaminant sources: Polychlorinated biphenyls and other organochlorines in bottom-dwelling animals from an Arctic estuary. *Sci Total Environ*, 160-61, 265-283.
Brown F.R., Winkler J., Visita P., Dhaliwal J. & Petreas M. (2006). Levels of PBDEs. PCDDs, PCDFs, and coplanar PCBs in edible fish from California coastal waters. *Chemosphere*, 64, 276-286.
Burger J., Gochfeld M., Jeitner C., Burke S. & Stamm T. (2007c). Metal levels in flathead sole (*Hippoglossoides elassodon*) and great sculpin (*Myoxocephalus polyacanthocephalus*) from Adak Island, Alaska: Potential risk to predators and fishermen. *Environ Res*, 103, 62-69.
Burger J., Gochfeld M., Jeitner C., Burke S., Stamm T., Snigaroff R., Snigaroff D., Patrick R. & Weston J. (2007b). Mercury levels and potential risk from subsistence foods from the Aleutians. *Sci Total Environ*, 384, 93-105.
Burger J., Gochfeld M., Shukla T., Jeitner C., Burke S., Donio M., Shukla S., Snigaroff R., Snigaroff D., Stamm T. & Volz C. (2007a). Heavy metals in Pacific Cod (*Gadus macrocephalus*) from the Aleutians: Location, age, size, and risk. *J Toxicol Env Heal A*, 70, 1897-1911.
Burger J., Jeitner C., Donio M., Shukla S. & Gochfeld M. (2009). Factors affecting mercury and selenium levels in New Jersey flatfish: Low risk to human consumers. *J Toxicol Env Heal A*, 72, 853-860.
Burger J., Jeitner C. & Gochfeld M. (2011). Locational differences in mercury and selenium levels in 19 species of saltwater fish from New Jersey. *J Toxicol Env Heal A*, 74, 863-874.

Burreau S., Zebuhr Y., Broman D. & Ishaq R. (2006). Biomagnification of PBDEs and PCBs in food webs from the Baltic Sea and the northern Atlantic ocean. *Sci Total Environ*, 366, 659-672.
Cai Y., Rooker J.R., Gill G.A. & Turner J.P. (2007). Bioaccumulation of mercury in pelagic fishes from the northern Gulf of Mexico. *Canadian Journal of Fisheries and Aquatic Sciences*, 64, 458-469.
Cakirogullari G.C., Kilic D. & Ucar Y. (2010). Levels of polychlorinated dibenzo-p-dioxins, dibenzo-p-furans and polychlorinated biphenyls in farmed sea bass (*Dicentrarchus labrax*) and sea bream (*Sparus aurata*) from Turkey. *Food Control*, 21, 1245-1249.
Cakirogullari G.C. & Secer S. (2010). Levels of DDTs and indicator polychlorinated biphenyls in Whiting (*Merlangius merlangus euxinus* N. 1840) and Horse mackerel (*Trachurus mediterraneus* S. 1868) from the Izmit Bay, Turkey. *Turkish Journal of Fisheries and Aquatic Sciences*, 10, 415-422.
Cakirogullari G.C., Ucar Y., Oymael B., Bozkurt E.N. & Kilic D. (2010). PCDD/F, dl-PCB and Indicator PCBs in Whiting, Horse Mackerel and Anchovy in Black Sea in Turkey. *Turkish Journal of Fisheries and Aquatic Sciences*, 10, 357-362.
Canedo-Lopez Y. & Macias-Zamora J.V. (2007). Polychlorinated dibenzo-p-dioxins and dibenzofurans in fish from four different regions of Mexico. *Ciencias Marinas*, 33, 217-227.
Capelli R., Contardi V., Cosma B., Minganti V. & Zanicchi G. (1983). A 4-year study on the distribution of some heavy metals in 5 marine organisms of the Ligurian Sea. *Marine Chemistry*, 12, 281-293.
Capelli R., Drava G., Siccardi C., De Pellegrini R. & Minganti V. (2004). Study of the distribution of trace elements in six species of marine organisms of the Ligurian Sea (north-western Mediterranean) - Comparison with previous findings. *Ann Chim-Rome*, 94, 533-546.
Capelli R., Minganti V. & Bernhard M. (1987). Total mercury, organic mercury, copper, manganese, selenium, and zinc in *Sarda sarda* from the Gulf of Genoa. *Sci Total Environ*, 63, 83-99.
Carlsson P., Herzke D., Wedborg M. & Gabrielsen G.W. (2011). Environmental pollutants in the Swedish marine ecosystem, with special emphasis on polybrominated diphenyl ethers (PBDE). *Chemosphere*, 82, 1286-1292.
Carubelli G., Fanelli R., Mariam G., Nichetti S., Crosa G., Calarnari D. & Fattore E. (2007). PCB contamination in farmed and wild sea bass (*Dicentrarchus labrax* L.) from a coastal wetland area in central Italy. *Chemosphere*, 68, 1630-1635.
Chen M.H., Chen C.Y., Chang S.K. & Huang S.W. (2007). Total and organic mercury concentrations in the white muscles of swordfish (*Xiphias gladius*) from the Indian and Atlantic oceans. *Food Addit Contam*, 24, 969-75.
Cheung K.C., Leung H.M. & Wong M.H. (2008). Metal concentrations of common freshwater and marine fish from the Pearl River Delta, South China. *Archives of Environmental Contamination and Toxicology*, 54, 705-715.
Chouvelon T., Warnau M., Churlaud C. & Bustamante P. (2009). Hg concentrations and related risk assessment in coral reef crustaceans, molluscs and fish from New Caledonia. *Environ. Pollut.*, 157, 331-340.
Cipro C.V.Z., Colabuono F.I., Taniguchi S. & Montone R.C. (2013). Persistent organic pollutants in bird, fish and invertebrate samples from King George Island, Antarctica. *Antarctic Science*, 25, 545-552.
Coelhan M., Strohmeier J. & Barlas H. (2006). Organochlorine levels in edible fish from the Marmara Sea, Turkey. *Environment International*, 32, 775-780.

Collings S.E., Johnson M.S. & Leah R.T. (1996). Metal contamination of angler-caught fish from the Mersey Estuary. *Marine Environmental Research*, 41, 281-297.

Contardi V., Capelli R., Pellacani T. & Zanicchi G. (1979). PCBs and chlorinated pesticides in organisms from the Ligurian Sea. *Mar Pollut Bull*, 10, 307-311.

Cornish A.S., Ng W.C., Ho V.C.M., Wong H.L., Lam J.C.W., Lam P.K.S. & Leung K.M.Y. (2007). Trace metals and organochlorines in the bamboo shark *Chiloseyllium plagiosum* from the southern waters of Hong Kong, China. *Sci Total Environ*, 376, 335-345.

Corsolini S., Ademollo N., Mariottini M., Fossi S., Guerranti C., Perra G., Duhamel G. & Focardi S. (2002). Polychlorinated biphenyls, polychlorinated-dibenzodioxins and -dibenzofurans in mackerel icefish and marbled rockcod from the Kerguelen Islands (Antarctica). *Organohalogen Compounds*, 57, 161-164.

Corsolini S., Ademollo N., Romeo T., Olmastroni S. & Focardi S. (2003). Persistent organic pollutants in some species of a Ross Sea pelagic trophic web. *Antarctic Science*, 15, 95-104.

Corsolini S., Focardi S., Kannan K., Tanabe S., Borrell A. & Tatsukawa R. (1995). Congener profile and toxicity assessment of polychlorinated biphenyls in dolphins, sharks, and tuna collected from Italian coastal waters. *Marine Environmental Research*, 40, 33-53.

Corsolini S., Guerranti C., Perra G. & Focardi S. (2008). Polybrominated diphenyl ethers, perfluorinated compounds and chlorinated pesticides in swordfish (*Xiphias gladius*) from the Mediterranean Sea. *Environ Sci Technol*, 42, 4344-4349.

Corsolini S., Romeo T., Ademolla N., Greco S. & Focardi S. (2002). POPs in key species of marine Antarctic ecosystem. *Microchemical Journal*, 73, 187-193.

Cronin M., Davies I.M., Newton A., Pirie J.M., Topping G. & Swan S. (1998). Trace metal concentrations in deep sea fish from the North Atlantic. *Marine Environmental Research*, 45, 225-238.

Cross F.A., Hardy L.H., Jones N.Y. & Barber R.T. (1973). Relation between total-body weight and concentrations of manganese, iron, copper, zinc, and mercury in white muscle of bluefish (*Pomatomus saltatrix*) and a bathyl-demersal fish *Antimora rostrata*. *J Fish Res Board Can*, 30, 1287-1291.

Cullon D.L., Yunker M.B., Alleyne C., Dangerfield N.J., O'Neill S., Whiticar M.J. & Ross P.S. (2009). Persistent organic pollutants in chinook salmon (*Oncorhynchus tshawytscha*): Implications for resident killer whales of British Columbia and adjacent waters. *Environ Toxicol Chem*, 28, 148-161.

Cutshall N.H., Naidu J.R. & Pearcy W.G. (1978). Mercury concentrations in Pacific hake, *Merluccius Productus* (Ayres), as a function of length and latitude. *Science*, 200, 1489-1491.

da Silva A.M.F., Lemes V.R.R., Barretto H.H.C., Oliveira E.S., de Alleluia I.B. & Paumgartten F.J.R. (2003). Polychlorinated biphenyls and organochlorine pesticides in edible fish species and dolphins from Guanabara Bay, Rio de Janeiro, Brazil. *Bulletin of Environmental Contamination and Toxicology*, 70, 1151-1157.

Davodi M., Esmaili-Sari A. & Bahramifarr N. (2011). Concentration of polychlorinated biphenyls and organochlorine pesticides in some edible fish species from the Shadegan Marshes (Iran). *Ecotoxicology and Environmental Safety*, 74, 294-300.

de Azevedo e Silva C.E., Azeredo A., Lima Dias A.d.C., Costa P., Lailson-Brito J., Malm O., Davee Guimaraes J.R. & Machado Torres J.P. (2009). Organochlorine compounds in sharks from the Brazilian coast. *Mar Pollut Bull*, 58, 294-298.

de Brito A.P.X., Takahashi S., Ueno D., Iwata H., Tanabe S. & Kubodera T. (2002). Organochlorine and butyltin residues in deep-sea organisms collected from the western North Pacific, off-Tohoku, Japan. *Mar Pollut Bull*, 45, 348-361.

De Marco S.G., Botte S.E. & Marcovecchio J.E. (2006). Mercury distribution in abiotic and biological compartments within several estuarine systems from Argentina: 1980-2005 period. *Chemosphere*, 65, 213-223.

de Mora S., Fowler S.W., Tolosa I., Villeneuve J.P. & Cattini C. (2005). Chlorinated hydrocarbons in marine biota and coastal sediments from the Gulf and Gulf of Oman. *Mar Pollut Bull*, 50, 835-849.

de Pinho A.P., Guimaraes J.R.D., Martins A.S., Costa P.A.S., Olavo G. & Valentin J. (2002). Total mercury in muscle tissue of five shark species from Brazilian offshore waters: Effects of feeding habit, sex, and length. *Environ Res*, 89, 250-258.

Delval C., Fournier S. & Vigneault Y. (1986). Polychlorinated biphenyl residues in some marine organisms from the Baie des Anglais (Baie-Comeau, Quebec, Saint-Lawrence Estuary). *Bulletin of Environmental Contamination and Toxicology*, 37, 823-829.

Denton G.R.W., Concepcion L.P., Wood H.R. & Morrison R.J. (2006). Polychlorinated biphenyls (PCBs) in marine organisms from four harbours in Guam. *Mar Pollut Bull*, 52, 214-226.

Deshpande A., Bhendigeri S., Shirsekar T., Dhaware D. & Khandekar R.N. (2009). Analysis of heavy metals in marine fish from Mumbai Docks. *Environ Monit Assess*, 159, 493-500.

Deshpande A.D., Dockum B.W., Cleary T., Farrington C. & Wieczorek D. (2013). Bioaccumulation of polychlorinated biphenyls and organochlorine pesticides in young-of-the-year bluefish (*Pomatomus saltatrix*) in the vicinity of a Superfund Site in New Bedford Harbor, Massachusetts, and in the adjacent waters. *Mar Pollut Bull*, 72, 146-164.

Deshpande A.D., Draxler A.F., Zdanowicz V.S., Schrock M.E. & Paulson A.J. (2000). Contaminant levels in the muscle of four species of fish important to the recreational fishery of the New York Bight Apex. *Mar Pollut Bull*, 44, 164-71.

Dewailly E., Rouja P., Dallaire R., Pereg D., Tucker T., Ward J., Weber J.P., Maguire J.S. & Julien P. (2008). Balancing the risks and the benefits of local fish consumption in Bermuda. *Food Addit Contam A*, 25, 1328-1338.

Di Bella G., Licata P., Bruzzese A., Naccari C., Trombetta D., Lo Turco V., Dugo G., Richetti A. & Naccari F. (2006). Levels and congener pattern of polychlorinated biphenyl and organochlorine pesticide residues in bluefin tuna (*Thunnus thynnus*) from the Straits of Messina (Sicily, Italy). *Environment International*, 32, 705-710.

Di Muccio A., Stefanelli P., Funari E., Barbini A., Generali T., Pelosi P., Girolimetti S., Amendola G., Vanni F. & Di Muccio S. (2002). Organochlorine pesticides and polychlorinated biphenyl in 12 edible marine organisms from the Adriatic Sea, Italy, Spring 1997. *Food Addit Contam*, 19, 1148-1161.

Dierking J., Wafo E., Schembri T., Lagadec V., Nicolas C., Letourneur Y. & Harmelin-Vivien M. (2009). Spatial patterns in PCBs, pesticides, mercury and cadmium in the common sole in the NW Mediterranean Sea, and a novel use of contaminants as biomarkers. *Mar Pollut Bull*, 58, 1605-14.

Easton M.D.L., Luszniak D. & Von der Geest E. (2002). Preliminary examination of contaminant loadings in farmed salmon, wild salmon and commercial salmon feed. *Chemosphere*, 46, 1053-1074.

Elhamri H., Idrissi L., Coquery M., Azemard S., El Abidi A., Benlemlih M., Saghi M. & Cubadda F. (2007). Hair mercury levels in relation to fish consumption in a community of the Moroccan Mediterranean coast. *Food Addit Contam*, 24, 1236-1246.

Endo T., Minoshima Y., Hisamichi Y., Kimura O., Hayasaka M., Ogasawara H. & Haraguchi K. (2012). Levels of mercury and organohalogen compounds in the muscle and liver of Kidako moray eels (*Gymnothorax kidako*) caught off the southern region of Japan. *Biological & Pharmaceutical Bulletin*, 35, 1745-1751.

Ernst W., Goerke H., Eder G. & Schaefer R.G. (1976). Residues of chlorinated hydrocarbons in marine organisms in relation to size and ecological parameters. I. PCB, DDT, DDE, and DDD in fishes and mollusks from the English Channel. *Bulletin of Environmental Contamination and Toxicology*, 15, 55-65.

Escobar-Sanchez O., Galvan-Magana F. & Rosiles-Martinez R. (2010). Mercury and selenium bioaccumulation in the smooth hammerhead shark, *Sphyrna zygaena linnaeus*, from the Mexican Pacific Ocean. *Bulletin of Environmental Contamination and Toxicology*, 84, 488-491.

Evans D.W. & Crumley P.H. (2005). Mercury in Florida Bay fish: Spatial distribution of elevated concentrations and possible linkages to Everglades restoration. *B Mar Sci*, 77, 321-345.

Fairey R., Taberski K., Lamerdin S., Johnson E., Clark R.P., Downing J.W., Newman J. & Petreas M. (1997). Organochlorines and other environmental contaminants in muscle tissues of sportfish collected from San Francisco Bay. *Mar Pollut Bull*, 34, 13.

Fairey R., Taberski K., Lamerdin S., Johnson E., Clark R.P., Downing J.W., Newman J. & Petreas M. (1997). Organochlorines and other environmental contaminants in muscle tissues of sportfish collected from San Francisco Bay. *Mar Pollut Bull*, 34, 1058-1071.

Falandysz J. (1984). Organochlorine pesticides and polychlorinated biphenyls in herring from the southern Baltic, 1981. *Zeitschrift Fur Lebensmittel-Untersuchung Und-Forschung*, 179, 20-23.

Falandysz J. (1984). Organochlorine pesticides and polychlorinated biphenyls in sprats from the southern Baltic, 1981. *Zeitschrift Fur Lebensmittel-Untersuchung Und-Forschung*, 178, 461-464.

Falandysz J. (1985). Organochlorine pesticides and polychlorinated biphenyls in cod from the southern Baltic, 1981. *Zeitschrift Fur Lebensmittel-Untersuchung Und-Forschung*, 181, 316-317.

Falandysz J. (1985). Organochlorine pesticides and polychlorinated biphenyls in flatfish from the southern Baltic, 1983. *Zeitschrift Fur Lebensmittel-Untersuchung Und-Forschung*, 181, 370-374.

Falandysz J. (1986). Organochlorine pesticides and polychlorinated bipheyls in cod from southern Baltic, 1983. *Zeitschrift Fur Lebensmittel-Untersuchung Und-Forschung*, 182, 136-139.

Falandysz J. (1986). Organochlorine pesticides and polychlorinated bipheyls in herring from southern Baltic, 1983. *Zeitschrift Fur Lebensmittel-Untersuchung Und-Forschung*, 182, 131-135.

Falandysz J., Wyrzykowska B., Puzyn T., Strandberg L. & Rappe C. (2002). Polychlorinated biphenyls (PCBs) and their congener-specific accumulation in edible fish from the Gulf of Gdansk, Baltic Sea. *Food Addit Contam*, 19, 779-795.

Fang J.K.H., Wu R.S.S., Zheng G.J., Au D.W.T., Lam P.K.S. & Shin P.K.S. (2009). The use of muscle burden in rabbitfish *Siganus oramin* for monitoring polycyclic aromatic hydrocarbons and polychlorinated biphenyls in Victoria Harbour, Hong Kong and potential human health risk. *Sci Total Environ*, 407, 4327-4332.

Ferreira A.G., Faria V.V., de Carvalho C.E.V., Lessa R.P.T. & da Silva F.M.S. (2004). Total mercury in the night shark, *Carcharhinus signatus* in the western equatorial Atlantic Ocean. *Braz Arch Biol Techn*, 47, 629-634.

Ferreira M., Antunes P., Costa J., Amado J., Gil O., Pousao-Ferreira P., Vale C. & Reis-Henriques M.A. (2008). Organochlorine bioaccumulation and biomarkers levels in culture and wild white seabream (*Diplodus sargus*). *Chemosphere*, 73, 1669-1674.

Focardi S., Lari L. & Marsili L. (1992). PCB-congeners, DDTs and hexachlorobenzene in Antarctic fish from Terra Nova Bay (Ross Sea). *Antarctic Science*, 4, 151-154.

Freeman H.C. & Horne D.A. (1973). Sampling edible muscle of swordfish (*Xiphias Gladius*) for total mercury analysis. *J Fish Res Board Can*, 30, 1251-1252.

Froescheis O., Looser R., Cailliet G.M., Jarman W.M. & Ballschmiter K. (2000). The deep-sea as a final global sink of semivolatile persistent organic pollutants? Part I: PCBs in surface and deep-sea dwelling fish of the North and South Atlantic and the Monterey Bay Canyon (California). *Chemosphere*, 40, 651-660.

García-Hernández J., Cadena-Cárdenas L., Betancourt-Lozano M., García-De-La-Parra L.M., García-Rico L. & Márquez-Farías F. (2007). Total mercury content found in edible tissues of top predator fish from the Gulf of California, Mexico. *Toxicological & Environmental Chemistry*, 89, 507-522.

Gassel M., Harwani S., Park J.-S. & Jahn A. (2013). Detection of nonylphenol and persistent organic pollutants in fish from the North Pacific Central Gyre. *Mar Pollut Bull*, 73, 231-242.

Gelsleichter J., Manire C.A., Szabo N.J., Cortes E., Carlson J. & Lombardi-Carlson L. (2005). Organochlorine concentrations in bonnethead sharks (*Sphyrna tiburo*) from four Florida estuaries. *Archives of Environmental Contamination and Toxicology*, 48, 474-483.

Geyer H., Freitag D. & Korte F. (1984). Polychlorinated biphenyls (PCBs) in the marine environment, particularly in the Mediterranean. *Ecotoxicology and Environmental Safety*, 8, 129-151.

Giam C.S., Chan H.S. & Neff G.S. (1978). Phthalate ester plasticizers, DDT, DDE, and polychlorinated biphenyls in biota from Gulf of Mexico. *Mar Pollut Bull*, 9, 249-251.

Gilmartin M. & Revelante N. (1975). Concentration of mercury, copper, nickel, silver, cadmium, and lead in Northern Adriatic anchovy, *Engraulis encrasicholus*, and sardine, *Sardinap pilchardus*. *Fish B-Noaa*, 73, 193-201.

Gochfeld M., Burger J., Jeitner C., Donio M. & Pittfield T. (2012). Seasonal, locational and size variations in mercury and selenium levels in striped bass (*Morone saxatilis*) from New Jersey. *Environ Res*, 112, 8-19.

Goncalves da Silva S.F., Bruening I.M.R.A., Montone R.C., Taniguchi S., Cascaes M.J., Dias P.S., Lavandier R.C., Hauser-Davis R.A. & Moreira I. (2013). Polybrominated diphenyl ethers (PBDES) and polychlorinated biphenyls (PCBS) in mussels and two fish species from the estuary of the Guanabara Bay, southeastern Brazil. *Bulletin of Environmental Contamination and Toxicology*, 91, 261-266.

Gossett R., Puffer H.W., Arthur R.H.J. & Young D.R. (1983). DDT, PCB and benzo(a)pyrene levels in white croaker (*Genyonemus lineatus*) from Southern California. *Mar Pollut Bull*, 14, 6.

Goutte A., Chevreuil M., Alliot F., Chastel O., Cherel Y., Eleaume M. & Masse G. (2013). Persistent organic pollutants in benthic and pelagic organisms off Adelie Land, Antarctica. *Mar Pollut Bull*, 77, 82-89.

Green N.W. & Knutzen J. (2003). Organohalogens and metals in marine fish and mussels and some relationships to biological variables at reference localities in Norway. *Mar Pollut Bull*, 46, 362-374.
Greenfield B.K. & Allen R.M. (2013). Polychlorinated biphenyl spatial patterns in San Francisco Bay forage fish. *Chemosphere*, 90, 1693-1703.
Greenfield B.K., Davis J.A., Fairey R., Roberts C., Crane D. & Ichikawa G. (2005). Seasonal, interannual, and long-term variation in sport fish contamination, San Francisco Bay. *Sci Total Environ*, 336, 25-43.
Greenfield B.K. & Jahn A. (2010). Mercury in San Francisco Bay forage fish. *Environ. Pollut.*, 158, 2716-2724.
Guruge K.S. & Tanabe S. (2001). Contamination by persistent organochlorines and butyltin compounds in the west coast of Sri Lanka. *Mar Pollut Bull*, 42, 179-186.
Guruge K.S. & Tanabe S. (2004). Polychlorinated dibenzo-p-dioxins, dibenzofurans and dioxin-like biphenyls in biota from Sri Lankan coast. *Mar Pollut Bull*, 48, 1004-1008.
Gutierrez-Mejia E., Lares M.L. & Sosa-Nishizaki O. (2009). Mercury and arsenic in muscle and liver of the golden cownose ray, *Rhinoptera steindachneri*, Evermann and Jenkins, 1891, from the Upper Gulf of California, Mexico. *Bulletin of Environmental Contamination and Toxicology*, 83, 230-234.
Hammerschmidt C.R. & Fitzgerald W.F. (2006). Bioaccumulation and trophic transfer of methylmercury in Long Island Sound. *Archives of Environmental Contamination and Toxicology*, 51, 416-424.
Hardell S., Tilander H., Welfinger-Smith G., Burger J. & Carpenter D.O. (2010). Levels of polychlorinated biphenyls (PCBs) and three organochlorine pesticides in fish from the Aleutian Islands of Alaska. *Plos One*, 5.
Harding G., Dalziel J. & Vass P. (2004). Prevalence and bioaccumilation of methylmercury in the food web of the Bay of Fundy, Gulf of Maine. In: *6th Bay of Fundy Workshop* (eds. Percy J, Evans A, Wells P & Rolston S). Environment Canada Nova Scotia, pp. 76-77.
Harding G.C., LeBlanc R.J., Vass W.P., Addison R.F., Hargrave B.T., Pearre S., Dupuis A. & Brodie P.F. (1997). Bioaccumulation of polychlorinated biphenyls (PCBs) in the marine pelagic food web, based on a seasonal study in the southern Gulf of St. Lawrence, 1976-1977. *Marine Chemistry*, 56, 145-179.
Harmelin-Vivien M., Bodiguel X., Charmasson S., Loizeau V., Mellon-Duval C., Tronczynski J. & Cossa D. (2012). Differential biomagnification of PCB, PBDE, Hg and radiocesium in the food web of the European hake from the NW Mediterranean. *Mar Pollut Bull*, 64, 974-983.
Heintz R., Krahn M.M., Vlitalo G.M. & Morado F. (2006). *Organochlorines in walleye pollock from the Bering Sea and Southeastern Alaska*.
Hellou J., Fancey L.L. & Payne J.F. (1992a). Concentrations of 24 elements in bluefin tuna, *Thunnus thynnus* from the northwest Atlantic. *Chemosphere*, 24, 211-218.
Hellou J., Warren W.G., Payne J.F., Belkhode S. & Lobel P. (1992b). Heavy metals and other elements in 3 tissues of cod, *Gadus morhua* from the northwest Atlantic. *Mar Pollut Bull*, 24, 452-458.
Hermanussen S., Matthews V., Paepke O., Limpus C.J. & Gaus C. (2008). Flame retardants (PBDEs) in marine turtles, dugongs and seafood from Queensland, Australia. *Mar Pollut Bull*, 57, 409-418.
Hisamichi Y., Haraguchi K. & Endo T. (2012). Levels of mercury and oranohalogen cmpounds in Pacific buefin tna (*Thunnus orientalis*) cltured in dfferent rgions of Japan. *Archives of Environmental Contamination and Toxicology*, 62, 296-305.

Hoekstra P.F., O'Hara T.M., Fisk A.T., Borga K., Solomon K.R. & Muir D.C.G. (2003). Trophic transfer of persistent orgranochlorine contaminants (OCs) within an Arctic marine food web from the southern Beaufort-Chukchi Seas. *Environ. Pollut.*, 124, 509-522.
Holden A., She J., Tanner M., Lunder S., Sharp R.e. & Hooper K. (2003). PBDEs in the San Francisco Bay area: Measurements in fish. *Organohalogen Compounds*, 61, 4.
Hong C.S., Bush B. & Xiao J. (1992). Coplanar PCBs in fish and mussels from marine and estuarine waters of New York state. *Ecotoxicology and Environmental Safety*, 23, 118-131.
Hueter R.E., Fong W.G., Henderson G., French M.F. & Manire C.A. (1995). Methylmercury concentration in shark muscle by species, size and distribution of sharks in Florida coastal waters. *Water Air and Soil Pollution*, 80, 893-899.
Ikemoto T., Tu N.P.C., Watanabe M.X., Okuda N., Omori K., Tanabe S., Tuyen B.C. & Takeuchi I. (2008). Analysis of biomagnification of persistent organic pollutants in the aquatic food web of the Mekong Delta, South Vietnam using stable carbon and nitrogen isotopes. *Chemosphere*, 72, 104-114.
Ikonomou M.G., Teas H.J., Gerlach R., Higgs D. & Addison R.F. (2011). Residues of PBDEs in northeastern Pacific marine fish: Evidence for spatial and temporal trends. *Environ Toxicol Chem*, 30, 1261-1271.
Impellizzeri G., Tringali C., Chillemi R. & Piattelli M. (1982). Observations on the levels of DDTs and PCBs in the central Mediterranean. *Sci Total Environ*, 25, 169-179.
Jacobs M.N., Covaci A. & Schepens P. (2002). Investigation of selected persistent organic pollutants in farmed Atlantic salmon (*Salmo salar*), salmon aquaculture feed, and fish oil components of the feed. *Environ Sci Technol*, 36, 2797-2805.
Jaeger I., Hop H. & Gabrielsen G.W. (2009). Biomagnification of mercury in selected species from an Arctic marine food web in Svalbard. *Sci Total Environ*, 407, 4744-51.
Jardine L.B., Burt M.D.B., Arp P.A. & Diamond A.W. (2009). Mercury comparisons between farmed and wild Atlantic salmon (*Salmo salar* L.) and Atlantic cod (*Gadus morhua* L.). *Aquac Res*, 40, 1148-1159.
Jarvis E., Schiff K., Sabin L. & Allen M.J. (2007). Chlorinated hydrocarbons in pelagic forage fishes and squid of the Southern California Bight. *Environ Toxicol Chem*, 26, 2290-2298.
Johnson L.L., Ylitalo G.M., Arkoosh M.R., Kagley A.N., Stafford C., Bolton J.L., Buzitis J., Anulacion B.F. & Collier T.K. (2007). Contaminant exposure in outmigrant juvenile salmon from Pacific Northwest estuaries of the United States. *Environ Monit Assess*, 124, 167-94.
Johnson-Restrepo B., Kannan K., Addink R. & Adams D.H. (2005). Polybrominated diphenyl ethers and polychlorinated biphenyls in a marine foodweb of coastal Florida. *Environ Sci Technol*, 39, 8243-8250.
Jothy A.A., Huschenbeth E. & Harms U. (1983). On the detection of heavy metals, organochlorine pesticides and polychlorinated biphenyls in fish and shellfish from the coastal waters of peninsular Malaysia. *Archiv Fur Fischereiwissenschaft*, 33, 161-206.
Julshamn K., Lundebye A.K., Heggstad K., Berntssen M.H.G. & Boe B. (2004). Norwegian monitoring programme on the inorganic and organic contaminants in fish caught in the Barents Sea, Norwegian Sea and North Sea, 1994-2001. *Food Addit Contam*, 21, 365-376.
Kannan K., Nakata H., Stafford R., Masson G.R., Tanabe S. & Giesy J.P. (1998). Bioaccumulation and toxic potential of extremely hydrophobic polychlorinated biphenyl congeners in biota collected at a superfund site contaminated with Aroclor 1268. *Environ Sci Technol*, 32, 1214-1221.

Kannan K., Smith R.G.J., Lee R.F., Windom H.L.H., P. T., Macauley J.M. & Summers J.K. (1998
). Distribution of total mercury and methylmercury in water, sediment, and fish
from south Florida estuaries. *Archives of Environmental Contamination and Toxicology*, 34, 109-118.
Kannan N., Choi H.K., Hong S.H., Oh J.R. & Shim W.J. (2010). Occurrence and biological fate of persistent organic contaminants in Yellow Sea fish. *EnvironmentAsia*, 3, 20-31.
Karouna-Renier N.K., Snyder R.A., Lange T., Gibson S., Allison J.G., Wagner M.E. & Ranga Rao K. (2011). Largemouth bass (*Micropterus salmoides*) and striped mullet (*Mugil cephalus*) as vectors of contaminants to human consumers in northwest Florida. *Marine Environmental Research*, 72, 96-104.
Kehrig H.D.A., Costa M., Moreira I. & Malm O. (2001). Methylmercury and total mercury in estuarine organisms from Rio de Janeiro, Brazil. *Environ Sci Pollut R*, 8, 275-279.
Kelly B.C., Gray S.L., Ikonomou M.G., MacDonald J.S., Bandiera S.M. & Hrycay E.G. (2007). Lipid reserve dynamics and magnification of persistent organic pollutants in spawning sockeye salmon (*Oncorhynchus nerka*) from the Fraser River, British Columbia. *Environ Sci Technol*, 41, 3083-3089.
Kelly B.C., Ikonomou M.G., Blair J.D. & Gobas F.A.P.C. (2008). Bioaccumulation behaviour of polybrominated diphenyl ethers (PBDEs) in a Canadian Arctic marine food web. *Sci Total Environ*, 401, 60-72.
Kennish M.J. & Ruppel B.E. (1996). Polychlorinated biphenyl contamination in selected estuarine and coastal marine finfish and shellfish of New Jersey. *Estuaries*, 19, 288-295.
Kennish M.J. & Ruppel B.E. (1998). Organochlorine contamination in selected estuarine and coastal marine finfish and shellfish of New Jersey. *Water Air and Soil Pollution*, 101, 123-136.
Khoshnoud M., Mobinia K., Javidniab K., Hosseinkhezric P. & Jamshidc K. (2011). Heavy Metals (Zn, Cu, Pb, Cd and Hg) Contents and Fatty Acids Ratios in Two Fish Species (*Scomberomorus commerson* and *Otolithes ruber*) of the Persian Gulf. *Iranian Journal of Pharmaceutical Sciences* 7, 191-196.
Koenig S., Huertas D. & Fernandez P. (2013). Legacy and emergent persistent organic pollutants (POPs) in NW Mediterranean deep-sea organisms. *Sci Total Environ*, 443, 358-366.
Koistinen J., Kiviranta H., Ruokojarvi P., Parmanne R., Verta M., Hallikainen A. & Vartiainen T. (2008). Organohalogen pollutants in herring, from the northern Baltic Sea: Concentrations, congener profiles, and explanatory factors. *Environ. Pollut.*, 154, 172-183.
Kojadinovic J., Potier M., Le Corre M., Cosson R.P. & Bustamante P. (2006). Mercury content in commercial pelagic fish and its risk assessment in the Western Indian Ocean. *Sci Total Environ*, 366, 688-700.
Kojadinovic J., Potier M., Le Corre M., Cosson R.P. & Bustamante P. (2007). Bioaccumulation of trace elements in pelagic fish from the Western Indian Ocean. *Environ. Pollut.*, 146, 548-566.
Kraepiel A.M., Keller K., Chin H.B., Malcolm E.G. & Morel F.M. (2003). Sources and variations of mercury in tuna. *Environ Sci Technol*, 37, 5551-8.
Kueh C.S.W. & Lam J.Y.C. (2008). Monitoring of toxic substances in the Hong Kong marine environment. *Mar Pollut Bull*, 57, 744-757.
Kumar M., Aalbersberg B. & Mosley L. (2004). Mercury levels in Fijian seafoods and
potential health implications: Report for World Health Organization. In.
Kutter V.T., Mirlean N., Baisch P.R.M., Kutter M.T. & Silva E. (2009). Mercury in freshwater, estuarine, and marine fishes from Southern Brazil and its ecological implication. *Environ Monit Assess*, 159, 35-42.

Kuzyk Z.A., Stow J.P., Burgess N.M., Solomon S.M. & Reimer K.J. (2005). PCBs in sediments and the coastal food web near a local contaminant source in Saglek Bay, Labrador. *Sci Total Environ*, 351, 264-284.

Kwasniak J., Falkowska L. & Kwasniak M. (2012). The assessment of organic mercury in Baltic fish by use of an in vitro digestion model. *Food Chem*, 132, 752-758.

Leah R.T., Johnson M.S., Connor L. & Levene C. (1997). Polychlorinated biphenyls in fish and shellfish from the Mersey Estuary and Liverpool Bay. *Marine Environmental Research*, 43, 345-358.

Lee J.S., Tanabe S., Takemoto N. & Kubodera T. (1997). Organochlorine residues in deep-sea organisms from Suruga Bay, Japan. *Mar Pollut Bull*, 34, 250-258.

Li Q., Yan C., Luo Z. & Zhang X. (2010). Occurrence and levels of polybrominated diphenyl ethers (PBDEs) in recent sediments and marine organisms from Xiamen offshore areas, China. *Mar Pollut Bull*, 60, 464-469.

Licata P., Trombetta D., Cristani M., Naccari C., Martino D., Calo M. & Naccari F. (2005). Heavy metals in liver and muscle of bluefin tuna (*Thunnus thynnus*) caught in the straits of Messina (Sicily, Italy). *Environ Monit Assess*, 107, 239-248.

Linko R.R. & Terho K. (1977). Occurrence of methylmercury in pike and Baltic herring from Turku Archipelago. *Environ. Pollut.*, 14, 227-235.

Lo Turco V., Di Bella G., La Pera L., Conte F., Macri B. & mo Dugo G. (2007). Organochlorine pesticides and polychlorinated biphenyl residues in reared and wild *Dicentrarchus labrax* from the Mediterranean Sea (Sicily, Italy). *Environ Monit Assess*, 132, 411-417.

Losada S., Roach A., Roosens L., Santos F.J., Galceran M.T., Vetter W., Neels H. & Covaci A. (2009). Biomagnification of anthropogenic and naturally-produced organobrominated compounds in a marine food web from Sydney Harbour, Australia. *Environment International*, 35, 1142-1149.

Luckhurst B.E., Prince E.D., Llopiz J.K., Snodgrass D. & Brothers E.B. (2006). Evidence of blue marlin (*Makaira nigricans*) spawning in Bermuda waters and elevated mercury levels in large specimens. *B Mar Sci*, 79, 691-704.

Magalhaes M.C., Costa V., Menezes G.M., Pinho M.R., Santos R.S. & Monteiro L.R. (2007
). Intra- and inter-specific variability in total and methylmercury bioaccumulation by eight marine fish species from the Azores. *Mar Pollut Bull*, 1654-1661.

Marcovecchio J.E., Moreno V.J. & Perez A. (1986). Biomagnification of total mercury in Bahia-Blanca Estuary shark. *Mar Pollut Bull*, 17, 276-278.

Marcovecchio J.E., Moreno V.J. & Perez A. (1991). Metal accumulation in tissues of sharks from the Bahia Blanca Estuary, Argentina. *Marine Environmental Research*, 31, 263-274.

Marsico E.T., Machado M.E.S., Knoff M. & Clemente S.C.S. (2007). Total mercury in sharks along the southern Brazilian Coast. *Arq Bras Med Vet Zoo*, 59, 1593-1596.

Martinez-Gomez C., Fernandez B., Benedicto J., Valdes J., Campillo J.A., Leon V.M. & Vethaak A.D. (2012). Health status of red mullets from polluted areas of the Spanish Mediterranean coast, with special reference to Portman (SE Spain). *Marine Environmental Research*, 77, 50-59.

Masmoudi W., Romdhane M.S., Kheriji S. & El Cafsi M.h. (2007). Polychlorinated biphenyl residues in the golden grey mullet (*Liza aurata*) from Tunis Bay, Mediterranean sea (Tunisia). *Food Chem*, 105, 72-76.

Mason R.P., Heyes D. & Sveinsdottir A. (2006). Methylmercury concentrations in fish from tidal waters of the Chesapeake Bay. *Archives of Environmental Contamination and Toxicology*, 51, 425-437.

Matthews V., Papke O. & Gaus C. (2008). PCDD/Fs and PCBs in seafood species from Moreton Bay, Queensland, Australia. *Mar Pollut Bull*, 57, 392-402.
McArthur T., Butler E.C.V. & Jackson G.D. (2003). Mercury in the marine food chain in the Southern Ocean at Macquarie Island: an analysis of a top predator, Patagonian toothfish (*Dissostichus eleginoides*) and a mid-trophic species, the warty squid (*Moroteuthis ingens*). *Polar Biol*, 27, 1-5.
Meador J.P., Ernest D.W. & Kagley A.N. (2005). A comparison of the non-essential elements cadmium, mercury, and lead found in fish and sediment from Alaska and California. *Sci Total Environ*, 339, 189-205.
Menasveta P. & Siriyong R. (1977). Mercury content of several predacious fish in the Andaman sea. *Mar Pollut Bull*, 8, 5.
Mendez E., Giudice H., Pereira A., Inocente G. & Medina D. (2001). Total mercury content - Fish weight relationship in swordfish (*Xiphias gladius*) caught in the southwest Atlantic Ocean. *J Food Compos Anal*, 14, 453-460.
Meng X.-Z., Guo Y., Mai B.-X. & Zeng E.Y. (2009). Enantiomeric signatures of chiral organochlorine pesticides in consumer fish from South China. *J Agr Food Chem*, 57, 4299-4304.
Minganti V., Drava G., De Pellegrini R. & Siccardi C. (2010). Trace elements in farmed and wild gilthead seabream, *Sparus aurata*. *Mar Pollut Bull*, 60, 2022-5.
Mizukawa K., Takada H., Takeuchi I., Ikemoto T., Omori K. & Tsuchiya K. (2009). Bioconcentration and biomagnification of polybrominated diphenyl ethers (PBDEs) through lower-trophic-level coastal marine food web. *Mar Pollut Bull*, 58, 1217-1224.
Moisey J., Fisk A.T., Hobson K.A. & Norstrom R.J. (2001). Hexachlorocyclohexane (HCH) isomers and chiral signatures of alpha-HCH in the arctic marine food web of the Northwater Polynya. *Environ Sci Technol*, 35, 1920-1927.
Mol J.H., Ramlal J.S., Lietar C. & Verloo M. (2001). Mercury contamination in freshwater, estuarine, and marine fishes in relation to small-scale gold mining in Suriname, South America. *Environ Res*, 86, 183-197.
Mondon J.A., Nowak B.F. & Sodergren A. (2001). Persistent organic pollutants in oysters *Crassostrea gigas* and sand flathead *Platycephalus bassensis* from Tasmanian estuarine and coastal waters. *Mar Pollut Bull*, 42, 157-161.
Monirith I., Nakata H., Tanabe S. & Tana T.S. (1999). Persistent organochlorine residues in marine and freshwater fish in Cambodia. *Mar Pollut Bull*, 38, 604-612.
Monod J.L., Arnaud P.M. & Arnoux A. (1995). PCB congeners in the marine biota of Saint-Paul and Amsterdam islands, southern Indian Ocean. *Mar Pollut Bull*, 30, 272-274.
Monosson E., Ashley J.T.F., McElroy A.E., Woltering D. & Elskus A.A. (2003). PCB congener distributions in muscle, liver and gonad of *Fundulus heteroclitus* from the lower Hudson River Estuary and Newark Bay. *Chemosphere*, 52, 777-787.
Monteiro L.R. & Lopes H.D. (1990). Merucy content of swordfish, *Xiphias gladius*, in relation to length, weight, age, and sex. *Mar Pollut Bull*, 21, 293-296.
Muir D., Savinova T., Savinov V., Alexeeva L., Potelov V. & Svetochev V. (2003). Bioaccumulation of PCBs and chlorinated pesticides in seals, fishes and invertebrates from the White Sea, Russia. *Sci Total Environ*, 306, 111-131.
Munshi A.B., Boardman G.D., Flick G.J., Cobb J. & Lane R.M. (2009). Pesticides (OCPS) and polychlorinated biphenyls (PCBs) concentration in various fish species along the Chesapeake Bay near Virginia Beach on the Atlantic coastline. *Open Oceanography Journal*, 3, 1-7.

Munshi A.B., Hina A.S. & Usmani T.H. (2005). Determination of the level of PCBs in small fishes from three different coastal areas of Karachi, Pakistan. *Pakistan Journal of Scientific and Industrial Research*, 48, 247-251.

Nadal M., Ferre-Huguet N., Marti-Cid R., Schuhmacher M. & Domingo J.L. (2008). Exposure to metals through the consumption of fish and seafood by the population living near the Ebro River in Catalonia, Spain: Health risks. *Hum Ecol Risk Assess*, 14, 780-795.

Naito W., Jin H.C., Kang Y.S., Yamamuro M., Masunaga S. & Nakanishi J. (2003). Dynamics of PCDDs/DFs and coplanar-PCBs in an aquatic food chain of Tokyo Bay. *Chemosphere*, 53, 347-362.

Nesto N., Romano S., Moschino V., Mauri M. & Da Ros L. (2007). Bioaccumulation and biomarker responses of trace metals and micro-organic pollutants in mussels and fish from the Lagoon of Venice, Italy. *Mar Pollut Bull*, 55, 469-484.

Nfon E., Cousins I.T., Jarvinen O., Mukherjee A.B., Verta M. & Broman D. (2009). Trophodynamics of mercury and other trace elements in a pelagic food chain from the Baltic Sea. *Sci Total Environ*, 407, 6267-6274.

Nicholson G.J., Theodoropoulos T. & Fabris G.J. (1994). Hydrocarbons, pesticides, PCB and PAH in Port Phillip Bay (Victoria) sand flathead. *Mar Pollut Bull*, 28, 115-120.

Nie X.P., Lan C.Y., Wei T.L. & Yang Y.F. (2005). Distribution of polychlorinated biphenyls in the water, sediment and fish from the Pearl River estuary, China. *Mar Pollut Bull*, 50, 537-546.

Nisbet I.C.T. & Reynolds L.M. (1984). Organochlorine residues in common terns and associated estuarine organisms, Massachusetts, USA, 1971-81. *Marine Environmental Research*, 11, 33-66.

Okumura Y., Kohno Y., Kamiyama T., Suzuki T. & Yamashita Y. (2005). Dioxin concentrations in marbled sole collected from Sendai Bay, Japan. *Mer (Tokyo)*, 43, 75-87.

Okumura Y., Yamashita Y. & Isagawa S. (2004). Concentrations of polychlorinated dibenzo-p-dioxins, dibenzofurans, non-ortho polychlorinated biphenyls, and mono-ortho polychlorinated biphenyls in Japanese flounder, with reference to the relationship between body length and concentration. *J Environ Monitor*, 6, 201-208.

O'Neill S.M. & West J.E. (2009). Marine distribution, life history traits, and the accumulation of polychlorinated biphenyls in chinook salmon from Puget Sound, Washington. *Transactions of the American Fisheries Society*, 138, 616-632.

Orbea A., Ortiz-Zarragoitia M., Sole M., Porte C. & Cajaraville M.P. (2002). Antioxidant enzymes and peroxisome proliferation in relation to contaminant body burdens of PAHs and PCBs in bivalve molluscs, crabs and fish from the Urdaibai and Plentzia estuaries (Bay of Biscay). *Aquatic Toxicology*, 58, 75-98.

Paasivirta J. & Linko R. (1980). Environmental toxins in Finnish wildlife - A study on time trends of residue contents in fish during 1973-1978. *Chemosphere*, 9, 643-661.

Padula D.J., Daughtry B.J. & Nowak B.F. (2008). Dioxins, PCBs, metals, metalloids, pesticides and antimicrobial residues in wild and farmed Australian southern bluefin tuna (*Thunnus maccoyii*). *Chemosphere*, 72, 34-44.

Pandelova M., Henkelmann B., Roots O., Simm M., Jaerv L., Benfenati E. & Schramm K.W. (2008). Levels of PCDD/F and dioxin-like PCB in Baltic fish of different age and gender. *Chemosphere*, 71, 369-378.

Papetti P. & Rossi G. (2009). Heavy metals in the fishery products of low Lazio and the use of metallothionein as a biomarker of contamination. *Environ Monit Assess*, 159, 589-598.

Parera J., Abalos M., Santos F.J., Galceran M.T. & Abad E. (2013). Polychlorinated dibenzo-p-dioxins, dibenzofurans, biphenyls, paraffins and polybrominated diphenyl ethers in marine fish species from Ebro River Delta (Spain). *Chemosphere*, 93, 499-505.

Pastor A., Hernandez F., Peris M.A., Beltran J., Sancho J.V. & Castillo M.T. (1994). Levels of heavy metals in some marine organisms from the western Mediterranean area (Spain). *Mar Pollut Bull*, 28, 50-53.

Pastor D., Boix J., Fernandez V. & Albaiges J. (1996). Bioaccumulation of organochlorinated contaminants in three estuarine fish species (*Mullus barbatus, Mugil cephalus* and *Dicentrarcus labrax*). *Mar Pollut Bull*, 32, 257-262.

Paul M.C., Toia R.F. & von Nagy-Felsobuki E.I. (2003). A novel method for the determination of mercury and selenium in shark tissue using high-resolution inductively coupled plasma-mass spectrometry. *Spectrochim Acta B*, 58, 1687-1697.

Payne E.J. & Taylor D.L. (2010). Effects of diet composition and trophic structure on mercury bioaccumulation in temperate flatfishes. *Archives of Environmental Contamination and Toxicology*, 58, 431-443.

Pena-Abaurrea M., Weijs L., Ramos L., Borghesi N., Corsolini S., Neels H., Blust R. & Covaci A. (2009). Anthropogenic and naturally-produced organobrominated compounds in bluefin tuna from the Mediterranean Sea. *Chemosphere*, 76, 1477-1482.

Perttila M., Tervo V. & Parmanne R. (1982). Age dependence of the concentrations of harmful substances in Baltic herring (*Clupea harengus*). *Chemosphere*, 11, 1019-1026.

Perugini M., Cavaliere M., Giammarino A., Mazzone P., Olivieri V. & Amorena M. (2004). Levels of polychlorinated biphenyls and organochlorine pesticides in some edible marine organisms from the Central Adriatic Sea. *Chemosphere*, 57, 391-400.

Picer M., Picer N. & Ahel M. (1978). Chlorinated insecticide and PCB residues in fish and mussels of east coastal waters of the middle and north Adriatic sea, 1974-75. *Pesticides Monitoring Journal*, 12, 102-112.

Pinto B., Garritano S.L., Cristofani R., Ortaggi G., Giuliano A., Amodio-Cocchieri R., Cirillo T., De Giusti M., Boccia A. & Reali D. (2008). Monitoring of polychlorinated biphenyl contamination and estrogenic activity in water, commercial feed and farmed seafood. *Environ Monit Assess*, 144, 445-453.

Piraino M.N. & Taylor D.L. (2009). Bioaccumulation and trophic transfer of mercury in striped bass (*Morone saxatilis*) and tautog (*Tautoga onitis*) from the Narragansett Bay (Rhode Island, USA). *Marine Environmental Research*, 67, 117-128.

Polak-Juszczak L. (2009). Temporal trends in the bioaccumulation of trace metals in herring, sprat, and cod from the southern Baltic Sea in the 1994-2003 period. *Chemosphere*, 76, 1334-1339.

Porte C. & Albaiges J. (1994). Bioaccumulation patterns of hydrocarbons and polychlorinated biphenyls in bivalves, crustaceans, and fishes. *Archives of Environmental Contamination and Toxicology*, 26, 273-281.

Quinete N., Lavandier R., Dias P., Taniguchi S., Montone R. & Moreira I. (2011). Specific profiles of polybrominated diphenylethers (PBDEs) and polychlorinated biphenyls (PCBs) in fish and tucuxi dolphins from the estuary of Paraiba do Sul River, Southeastern Brazil. *Mar Pollut Bull*, 62, 440-446.

Ramu K., Kajiwara N., Mochizuki H., Miyasaka H., Asante K.A., Takahashi S., Ota S., Yeh H.M., Nishida S. & Tanabe S. (2006). Occurrence of organochlorine pesticides, polychlorinated biphenyls and

polybrominated diphenyl ethers in deep-sea fishes from the Sulu Sea. *Mar Pollut Bull*, 52, 1827-1832.

Ravid R., Benyosef J. & Hornung H. (1985). PCBs, DDTs and other chlorinated hydrocarbons in marine organisms from the Mediterranean coast of Israel. *Mar Pollut Bull*, 16, 35-38.

Rider S.J. & Adams D.H. (2000). Mercury concentration in spotted seatrout (*Cynoscion nebulosus*) from northwest Florida. *Gulf of Mexico Science*, 2, 97-103.

Riget F., Moller P., Dietz R., Nielsen T.G., Asmund G., Strand J., Larsen M.M. & Hobson K.A. (2007). Transfer of mercury in the marine food web of West Greenland. *J Environ Monitor*, 9, 877-883.

Rivers J.B., Pearson J.E. & Stultz C.D. (1972). Total and organic mercury in marine fish. *Bulletin of Environmental Contamination and Toxicology*, 3, 255-266.

Rochman C.M., Lewison R.L., Eriksen M., Allen H., Cook A.-M. & Teh S.J. (2014). Polybrominated diphenyl ethers (PBDEs) in fish tissue may be an indicator of plastic contamination in marine habitats. *Sci Total Environ*, 476, 622-633.

Romeo M., Siau Y., Sidoumou Z. & Gnassia-Barelli M. (1999). Heavy metal distribution in different fish species from the Mauritania coast. *Sci Total Environ*, 232, 169-175.

Roots O., Holoubek I. & Zitko V. (2003). Polychlorinated biphenyls and chlororganic pesticides patterns in perch (*Perca fluviatilis*). *Fresen Environ Bull*, 12, 883-900.

Ruelas-Inzunza J., Meza-Lopez G. & Paez-Osuna F. (2008). Mercury in fish that are of dietary importance from the coasts of Sinaloa (SE Gulf of California). *J Food Compos Anal*, 21, 211-218.

Ruelas-Inzunza J. & Paez-Osuna F. (2005). Mercury in fish and shark tissues from two coastal lagoons in the gulf of California, Mexico. *Bulletin of Environmental Contamination and Toxicology*, 74, 294-300.

Saei-Dehkordi S.S., Fallah A.A. & Nematollahi A. (2010). Arsenic and mercury in commercially valuable fish species from the Persian Gulf: Influence of season and habitat. *Food Chem Toxicol*, 48, 2945-2950.

Said T.O. (2007). Determination of persistent organic pollutants in sediment and fish of the western coast of Alexandria, Egypt. *Chemistry and Ecology*, 23, 289-302.

Sajwan K., Nune S., Richardson J., Senthilkumar K. & Loganathan B. (2006). Contamination profiles of persistent organochlorines and polybrominated diphenyl ethers in fish from coastal waters off Savannah, GA, USA. *Organohalogen Compounds*, 68, 4.

Salama A.A., Mohamed M.A.M., Duval B., Potter T.L. & Levin R.E. (1998). Polychlorinated biphenyl concentration in raw and cooked North Atlantic bluefish (*Pomatomus saltatrix*) fillets. *J Agr Food Chem*, 46, 1359-1362.

Sapota G. & Wisniewska-Wojtasik B. (2007). Persistent organic pollutant content in cod (*Gadus morhua* L.) from the Barents Sea region. *Oceanological and Hydrobiological Studies*, 36, 65-77.

Satsmadjis J. & Gabrielides G.P. (1979). Observations on the concentration levels of chlorinated hydrocarbons in a Mediterranean fish. *Mar Pollut Bull*, 10, 109-111.

Satsmadjis J., Georgakopoulosgregoriades E. & Voutsinoutaliadouri F. (1988). Red mullet contamination by PCBs and chlorinated pesticides in the Pagassitikos Gulf, Greece. *Mar Pollut Bull*, 19, 136-138.

Senn D.B., Chesney E.J., Blum J.D., Bank M.S., Maage A. & Shine J.P. (2010). Stable isotope (N, C, Hg) study of methylmercury sources and trophic transfer in the northern Gulf of Mexico. *Environ Sci Technol*, 44, 1630-1637.

Serrano R., Barreda M. & Blanes M.A. (2008). Investigating the presence of organochlorine pesticides and polychlorinated biphenyls in wild and farmed gilthead sea bream (*Sparus aurata*) from the Western Mediterranean sea. *Mar Pollut Bull*, 56, 963-972.
Shaw G.R. & Connell D.W. (1982). Factors influencing concentrations of polychlorinated biphenyls in organisms from an estuarine ecosystem. *Aust J Mar Fresh Res*, 33, 1057-1070.
Shaw S.D., Berger M.L., Brenner D., Kannan K., Lohmann N. & Paepke O. (2009). Bioaccumulation of polybrominated diphenyl ethers and hexabromocyclododecane in the northwest Atlantic marine food web. *Sci Total Environ*, 407, 3323-3329.
Shi J., Li Y., Liang H., Zheng G.J., Wu Y. & Liu W. (2013). OCPs and PCBs in marine edible fish and human health risk assessment in the eastern Guangdong, China. *Archives of Environmental Contamination and Toxicology*, 64, 632-642.
Shultz C.D. & Crear D. (1976). Distribution of total and organic mercury in 7 tissues of Pacific blue marlin, *Makaira nigricans*. *Pac Sci*, 30, 101-107.
Sivaperumal P., Sankar T. & Viswanathannair P. (2007). Heavy metal concentrations in fish, shellfish and fish products from internal markets of India vis-a-vis international standards. *Food Chem*, 102, 612-620.
Soto-Jimenez M.F., Amezcua F. & Gonzalez-Ledesma R. (2010). Nonessential metals in striped marlin and Indo-Pacific sailfish in the southeast Gulf of California, Mexico: Concentration and sssessment of human health risk. *Archives of Environmental Contamination and Toxicology*, 58, 810-818.
Staudinger M.D. (2011). Species- and size-specific variability of mercury concentrations in four commercially important finfish and their prey from the northwest Atlantic. *Mar Pollut Bull*, 62 734-740.
Stefanelli P., Ausili A., Ciuffa G., Colasanti A., Di Muccio S. & Morlino R. (2002). Investigation of polychlorobiphenyls and organochlorine pesticides in tissues of tuna (*Thunnus Thunnus thynnus*) from the Mediterranean Sea in 1999. *Bulletin of Environmental Contamination and Toxicology*, 69, 800-807.
Stefanelli P., Di Muccio A., Ferrara F., Barbini D.A., Generali T., Pelosi P., Amendola G., Vanni F., Di Muccio S. & Ausili A. (2004). Estimation of intake of organochlorine pesticides and chlorobiphenyls through edible fishes from the Italian Adriatic Sea during 1997. *Food Control*, 15, 27-38.
Steimle F.W., Zdanowicz V.S. & Gadbois D.F. (1990). Metals and organic contaminants in northwest Atlantic deep-sea tilefish tissues. *Mar Pollut Bull*, 21, 530-535.
Stephansen D.A., Svendsen T.C., Vorkamp K. & Frier J.-O. (2012). Changes in patterns of persistent halogenated compounds through a pelagic food web in the Baltic Sea. *Marine Environmental Research*, 73, 17-24.
Storelli M.M., Barone G. & Marcotrigiano G.O. (2007). Residues of polychlorinated biphenyls in edible fish of the Adriatic Sea: Assessment of human exposure. *Journal of Food Science*, 72, C183-C187.
Storelli M.M., Barone G., Piscitelli G. & Marcotrigiano G.O. (2007). Mercury in fish: concentration vs. fish size and estimates of mercury intake. *Food Addit Contam*, 24, 1353-7.
Storelli M.M., Ceci E., Storelli A. & Marcotrigiano G.O. (2003). Polychlorinated biphenyl, heavy metal and methylmercury residues in hammerhead sharks: contaminant status and assessment. *Mar Pollut Bull*, 46, 1035-1039.

Storelli M.M., Giacominelli Stuffler R. & Marcotrigiano G.O. (1998). Total mercury in muscle of benthic and pelagic fish from the South Adriatic Sea (Italy). *Food Addit Contam*, 15, 876-83.
Storelli M.M., Giacominelli-Stuffler R. & Marcotrigiano G.O. (2002). Total and methylmercury residues in cartilaginous fish from Mediterranean Sea. *Mar Pollut Bull*, 44, 1354-1358.
Storelli M.M., Giacominelli-Stuffler R., Storelli A., D'Addabbo R., Palermo C. & Marcotrigiano G.O. (2003). Survey of total mercury and methylmercury levels in edible fish from the Adriatic Sea. *Food Addit Contam*, 20, 1114-9.
Storelli M.M., Giacominelli-Stuffler R., Storelli A. & Marcotrigiano G.O. (2005). Accumulation of mercury, cadmium, lead and arsenic in swordfish and bluefin tuna from the Mediterranean Sea: A comparative study. *Mar Pollut Bull*, 50, 1004-1007.
Storelli M.M. & Marcotrigiano G.O. (2001). Persistent organochlorine residues and toxic evaluation of polychlorinated biphenyls in sharks from the Mediterranean Sea (Italy). *Mar Pollut Bull*, 42, 1323-1329.
Storelli M.M., Perrone V.G., Busco V.P., Spedicato D. & Barone G. (2012). Persistent Organic Pollutants (PCBs and DDTs) in European Conger Eel, Conger conger L., from the Ionian Sea (Mediterranean Sea). *Bulletin of Environmental Contamination and Toxicology*, 88, 928-932.
Storelli M.M., Stuffler R.G. & Marcotrigiano G.O. (2002). Total and methylmercury residues in tuna-fish from the Mediterranean sea. *Food Addit Contam*, 19, 715-720.
Strandberg B., Bandh C., van Bavel B., Bergqvist P.A., Broman D., Ishaq R., Naf C. & Rappe C. (2000). Organochlorine compounds in the Gulf of Bothnia: sediment and benthic species. *Chemosphere*, 40, 1205-1211.
Strid A., Athanassiadis I., Athanasiadou M., Svavarsson J., Papke O. & Bergman A. (2010). Neutral and phenolic brominated organic compounds of natural and anthropogenic origin in northeast Atlantic Greenland shark (*Somniosus microcephalus*). *Environ Toxicol Chem*, 29, 2653-2659.
Strom D.G. & Graves G.A. (2001). A comparison of mercury in estuarine fish between Florida Bay and the Indian River Lagoon, Florida, USA. *Estuaries*, 24, 597-609.
Suk S.H., Smith S.E. & Ramon D.A. (2009). Bioaccumulation of mercury in pelagic sharks from the northeast Pacific Ocean. *California Cooperative Oceanic Fisheries Investigations Reports*, 50, 172-177.
Sun Y.-X., Hao Q., Xu X.-R., Luo X.-J., Wang S.-L., Zhang Z.-W. & Mai B.-X. (2014). Persistent organic pollutants in marine fish from Yongxing Island, South China Sea: Levels, composition profiles and human dietary exposure assessment. *Chemosphere*, 98, 84-90.
Svendsen T.C., Vorkamp K., Ronsholdt B. & Frier J.-O. (2008). Retrospective determination of primary feeding areas of Atlantic salmon (*Salmo salar*) using fingerprinting of chlorinated organic contaminants. *Ices Journal of Marine Science*, 65, 921-929.
Szlinder-Richert J., Barska I., Mazerski J. & Usydus Z. (2008). Organochlorine pesticides in fish from the southern Baltic Sea: Levels, bioaccumulation features and temporal trends during the 1995-2006 period. *Mar Pollut Bull*, 56, 927-940.
Szlinder-Richert J., Barska I., Mazerski J. & Usydus Z. (2009). PCBs in fish from the southern Baltic Sea: Levels, bioaccumulation features, and temporal trends during the period from 1997 to 2006. *Mar Pollut Bull*, 58, 85-92.
Szlinder-Richert J., Barska I., Usydus Z., Ruczynska W. & Grabic R. (2009). Investigation of PCDD/Fs and dl-PCBs in fish from the southern Baltic Sea during the 2002-2006 period. *Chemosphere*, 74, 1509-1515.

Takahashi S., Lee J.S., Tanabe S. & Kubodera T. (1998). Contamination and specific accumulation of organochlorine and butyltin compounds in deep-sea organisms collected from Suruga Bay, Japan. *Sci Total Environ*, 214, 49-64.

Takahashi S., Oshihoi T., Ramu K., Isobe T., Ohmori K., Kubodera T. & Tanabe S. (2010). Organohalogen compounds in deep-sea fishes from the western North Pacific, off-Tohoku, Japan: Contamination status and bioaccumulation profiles. *Mar Pollut Bull*, 60, 187-196.

Takahashi S., Tanabe S. & Kawaguchi K. (2000). Organochlorine and butyltin residues in mesopelagic myctophid fishes from the western North Pacific. *Environ Sci Technol*, 34, 5129-5136.

Takeuchi I., Miyoshi N., Mizukawa K., Takada H., Ikemoto T., Omori K. & Tsuchiya K. (2009). Biomagnification profiles of polycyclic aromatic hydrocarbons, alkylphenols and polychlorinated biphenyls in Tokyo Bay elucidated by delta C-13 and delta N-15 isotope ratios as guides to trophic web structure. *Mar Pollut Bull*, 58, 663-671.

Tanabe S., Ramu K., Mochizuki H., Miyasaka H., Okuda N., Muraoka M., Kajiwara N., Takahashi S. & Kubodera T. (2005). Contamination and distribution of persistent organochlorine and organotin compounds in deep-sea organisms from East China Sea. *National Science Museum of Monographs*, 29, 24.

Thieleke J. (1973). Mercury levels in five species of commercially important pelagic fish taken from the Pacific Ocean near Hawaii. In. University of Wisconsin Madison, WI.

Tian S., Zhu L. & Liu M. (2010). Bioaccumulation and distribution of polybrominated diphenyl ethers in marine species from Bohai bay, China. *Environ Toxicol Chem*, 29, 2278-2285.

Trocino A., Xiccato G., Majolini D., Tazzoli M., Tulli F., Tibaldi E., Messina C.M. & Santulli A. (2012). Levels of dioxin-like polychlorinated biphenyls (DL-PCBs) and metals in European sea bass from fish farms in Italy. *Food Chem*, 134, 333-338.

Tyrell L., McHugh B., Glynn D., Twomey M., Joyce E., Costello J. & al. e. (2005). Trace metal concentrations in various fish species landed at selected Irish ports, 2003. In: *Marine Environment and Health Series* Abbotstown, Dublin.

Ueno D., Alaee M., Marvin C., Muir D.C.G., Macinnis G., Reiner E., Crozier P., Furdui V.I., Subramanian A., Fillmann G., Lam P.K.S., Zheng G.J., Muchtar M., Razak H., Prudente M., Chung K.-H. & Tanabe S. (2006). Distribution and transportability of hexabromocyclododecane (HBCD) in the Asia-Pacific region using skipjack tuna as a bioindicator. *Environ. Pollut.*, 144, 238-247.

Ueno D., Watanabe M., Subramanian A.L., Tanaka H., Fillmann G., Lam P.K.S., Zheng G.J., Muchtar M., Razak H., Prudente M., Chung K.H. & Tanabe S. (2005). Global pollution monitoring of polychlorinated dibenzo-p-dioxins (PCDDs), furans (PCDFs) and coplanar polychlorinated biphenyls (coplanar PCBs) using skipjack tuna as bioindicator. *Environ. Pollut.*, 136, 303-313.

Vandenbroek W.L.F. (1981). Concentration and distribution of mercury in flesh of orange roughy (*Hoplostethus atlanticus*). *New Zeal J Mar Fresh*, 15, 255-260.

Vassilopoulou V. & Georgakopoulosgregoriades E. (1993). Factors influencing the uptake of PCBs and DDTs in red mullet (Mullus barbatus) from Pagassitikos Gulf, central Greece. *Mar Pollut Bull*, 26, 285-287.

Viana F., Huertas R. & Danulat E. (2005). Heavy metal levels in fish from coastal waters of Uruguay. *Archives of Environmental Contamination and Toxicology*, 48, 530-537.

Villeneuve J.P., Fowler S.W. & Anderlini V.C. (1987). Organochlorine levels in edible marine organisms from Kuwaiti coastal waters. *Bulletin of Environmental Contamination and Toxicology*, 38, 266-270.

Voegborlo R.B., Matsuyama A., Akagi H., Adimado A.A. & Ephraim J.H. (2006). Total mercury and methylmercury accumulation in the muscle tissue of frigate (*Auxis thazard thazard*) and yellow fin (*Thunnus albacares*) tuna from the Gulf of Guinea, Ghana. *Bulletin of Environmental Contamination and Toxicology*, 76, 840-847.

Voorspoels S., Covaci A. & Schepens P. (2003). Polybrominated diphenyl ethers in marine species from the Belgian North Sea and the western Scheldt Estuary: Levels, profiles, and distribution. *Environ Sci Technol*, 37, 4348-4357.

Vorkamp K., Christensen J.H. & Riget F. (2004). Polybrominated diphenyl ethers and organochlorine compounds in biota from the marine environment of East Greenland. *Sci Total Environ*, 331, 143-155.

Vuorinen P.J., Keinanen M., Kiviranta H., Koistinen J., Kiljunen M., Myllyla T., Ponni J., Peltonen H., Verta M. & Karjalainen J. (2012). Biomagnification of organohalogens in Atlantic salmon (*Salmo salar*) from its main prey species in three areas of the Baltic Sea. *Sci Total Environ*, 421, 129-143.

Wan Y., Hu J., Zhang K. & An L. (2008). Trophodynamics of polybrominated diphenyl ethers in the marine food web of Bohai Bay, North China. *Environ Sci Technol*, 42, 1078-1083.

Wan Y., Jin X., Hu J. & Jin F. (2007). Trophic dilution of polycyclic aromatic hydrocarbons (PAHs) in a marine food web from Bohai Bay, North China. *Environ Sci Technol*, 41, 3109-3114.

Watling R.J., Mcclurg T.P. & Stanton R.C. (1981). Relation between mercury concentration and size in the mako shark. *Bulletin of Environmental Contamination and Toxicology*, 26, 352-358.

Webster L., Walsham P., Russell M., Hussy I., Neat F., Dalgarno E., Packer G., Scurfield J.A. & Moffat C.F. (2011). Halogenated persistent organic pollutants in deep water fish from waters to the west of Scotland. *Chemosphere*, 83, 839-850.

Xia C., Lam J.C.W., Wu X., Sun L., Xie Z. & Lam P.K.S. (2011). Levels and distribution of polybrominated diphenyl ethers (PBDEs) in marine fishes from Chinese coastal waters. *Chemosphere*, 82, 18-24.

Xiang C.H., Luo X.J., Chen S.J., Yu M., Mai B.X. & Zeng E.Y. (2007). Polybrominated diphenyl ethers in biota and sediments of the Pearl River Estuary, South China. *Environ Toxicol Chem*, 26, 616-623.

Yamashita Y., Omura Y. & Okazaki E. (2005). Total mercury and methylmercury levels in commercially important fishes in Japan. *Fisheries Sci*, 71, 1029-1035.

Ylitalo G.M., Buzitis J. & Krahn M.M. (1999). Analyses of tissues of eight marine species from Atlantic and Pacific coasts for dioxin-like chlorobiphenyls (CBs) and total CBs. *Archives of Environmental Contamination and Toxicology*, 37, 205-219.

Yu M., Luo X.-J., Wu J.-P., Chen S.J. & Mai B.-X. (2009). Bioaccumulation and trophic transfer of polybrominated diphenyl ethers (PBDEs) in biota from the Pearl River Estuary, South China. *Environment International*, 35, 1090-1095.

Zauke G.P., Savinov V.M., Ritterhoff J. & Savinova T. (1999). Heavy metals in fish from the Barents Sea in (summer 1994). *Sci Total Environ*, 227, 161-173.

Zhang K., Wan Y., An L. & Hu J. (2010). Trophodynamics of polybrominated diphenyl ethers and methoxylated polybrominated diphenyl ethers in a marine food web. *Environ Toxicol Chem*, 29, 2792-2799.

Zhang X.M., Naidu A.S., Kelley J.J., Jewett S.C., Dasher D. & Duffy L.K. (2001). Baseline concentrations of total mercury and methylmercury in salmon returning via the Bering Sea (1999-2000). *Mar Pollut Bull*, 42, 993-997.

Zhou S., Tong L., Tang Q., Gu X., Xue B. & Liu W. (2013). Residues, sources and tissue distributions of organochlorine pesticides in dog sharks (*Mustelus griseus*) from Zhoushan Fishing Ground, China. *Mar Pollut Bull*, 73, 374-380.
